# Quantitative analysis reveals crosstalk mechanisms of heat shock-induced attenuation of NF-κB signaling at the single cell level

**DOI:** 10.1371/journal.pcbi.1006130

**Published:** 2018-04-30

**Authors:** Małgorzata Kardyńska, Anna Paszek, Jarosław Śmieja, David Spiller, Wiesława Widłak, Michael R. H. White, Pawel Paszek, Marek Kimmel

**Affiliations:** 1 Systems Engineering Group, Silesian University of Technology, Gliwice, Poland; 2 System Microscopy Centre, School of Biological Sciences, Faculty of Biology, Medicine and Health, University of Manchester, Manchester Academic Health Science Centre, Manchester, United Kingdom; 3 Maria Skłodowska-Curie Institute–Oncology Center, Gliwice Branch, Gliwice, Poland; 4 Departments of Statistics and Bioengineering, Rice University, Houston, TX, United States of America; University of Virginia, UNITED STATES

## Abstract

Elevated temperature induces the heat shock (HS) response, which modulates cell proliferation, apoptosis, the immune and inflammatory responses. However, specific mechanisms linking the HS response pathways to major cellular signaling systems are not fully understood. Here we used integrated computational and experimental approaches to quantitatively analyze the crosstalk mechanisms between the HS-response and a master regulator of inflammation, cell proliferation, and apoptosis the Nuclear Factor κB (NF-κB) system. We found that populations of human osteosarcoma cells, exposed to a clinically relevant 43°C HS had an attenuated NF-κB p65 response to Tumor Necrosis Factor α (TNFα) treatment. The degree of inhibition of the NF-κB response depended on the HS exposure time. Mathematical modeling of single cells indicated that individual crosstalk mechanisms differentially encode HS-mediated NF-κB responses while being consistent with the observed population-level responses. In particular “all-or-nothing” encoding mechanisms were involved in the HS-dependent regulation of the IKK activity and IκBα phosphorylation, while others involving transport were “analogue”. In order to discriminate between these mechanisms, we used live-cell imaging of nuclear translocations of the NF-κB p65 subunit. The single cell responses exhibited “all-or-nothing” encoding. While most cells did not respond to TNFα stimulation after a 60 min HS, 27% showed responses similar to those not receiving HS. We further demonstrated experimentally and theoretically that the predicted inhibition of IKK activity was consistent with the observed HS-dependent depletion of the IKKα and IKKβ subunits in whole cell lysates. However, a combination of “all-or-nothing” crosstalk mechanisms was required to completely recapitulate the single cell data. We postulate therefore that the heterogeneity of the single cell responses might be explained by the cell-intrinsic variability of HS-modulated IKK signaling. In summary, we show that high temperature modulates NF-κB responses in single cells in a complex and unintuitive manner, which needs to be considered in hyperthermia-based treatment strategies.

## Author summary

Hyperthermia has been considered a promising strategy to sensitize cancer cells to treatment. As such, it might potentially increase treatment efficacy while reducing negative side effects. So far, this potential has not been fully realized. One of the major obstacles is a lack of quantitative understanding of crosstalk mechanisms involved in the heat shock and other cellular stress-factor systems. In this work, we used experimental population-level and live-cell imaging approaches to characterize the NF-κB signaling system response to physiologically high temperature. We then iteratively applied mathematical modeling of single cells to infer the underlying regulatory mechanisms. We showed experimentally that elevated temperature attenuated cytokine-induced NF-κB system responses, resulting in complex and unintuitive single cell behavior. Our analyses suggested that these responses are consistent with stochastic cell-intrinsic heat-shock-mediated regulation of the NF-κB system via the IKK signaling module. Considering the oncogenic associations of the NF-κB pathway, the crosstalk mechanisms described in this work are important for the understanding of chemo- and radio-resistance to treatment. However, more but similar, quantitative studies are required to ultimately change currently used therapy protocols.

## Introduction

Cells have developed complex mechanisms to counteract environmental stresses such as toxins, free radicals, and extreme temperatures [1]. These involve specific stress response pathways, which control genes and proteins responsible for survival and adaptation but also alter the regulation of other cellular systems, both at the single cell and at the cell population level. The latter may ultimately lead to disease states. For example, heat shock (HS) response confers thermotolerance preventing protein denaturation and apoptosis, and has been known to affect inflammatory and immune responses [2]. A quantitative understanding of the crosstalk between stress response pathways and other cellular systems in cells and cell populations could provide novel insights into disease pathology and new treatment strategies, perhaps involving the regulation of dynamic single cell decision-making and cellular heterogeneity.

Nuclear Factor κB (NF-κB) is a dynamic and versatile transcription factor (TF) that is widely involved in stress responses and the control of cell fate [3]. The NF-κB system is primarily responsible for regulation of inflammation and immune response (e.g. via regulation of cytokine production) and is also involved in apoptosis, cell cycle progression, angiogenesis, and metastasis by controlling context-dependent transcription of hundreds of different genes [4]. Up-regulation of the NF-κB pathway is frequently observed in cancer cells and this may contribute to their resistance to treatment (reviewed in [5]). NF-κB transcription factors are dimers formed by the members of the multigene NF-κB/Rel family, which in humans includes five proteins, but the RelA/p50 NF-κB1 is the most common, ubiquitously expressed heterodimer involved in the so-called canonical NF-κB signaling (involved in inflammation and response to cytokines etc., reviewed in [6]). In resting cells the NF-κB dimers are sequestered in the cytoplasm by association with Inhibitory κB (IκB) proteins, which mask the nuclear localization signals (NLSs) of NF-κB. Cytokine stimulation induces activation of the IκB kinase (IKK) complex, which in turn phosphorylates the IκB protein, leading to its proteasomal degradation. This allows the translocation of NF-κB to the nucleus and facilitates binding to the κB DNA regulatory elements (rev. in [7, 8]). The expression of IκBα is controlled by a highly NF-κB-responsive promoter [9], which together with the NF-κB-dependent A20 protein [10] generates negative feedback circuits that regulate the NF-κB response. Over the last decade, cutting-edge microscopy approaches have demonstrated that this two-feedback structure can support oscillations of the nuclear NF-κB in response to cytokine stimulation, including Tumor Necrosis Factor α (TNFα) [11, 12]. These analyses exemplify how complex environmental signals may be functionally encoded in the dynamical NF-κB system response [13], which is in part facilitated by crosstalk with other cellular signaling systems [14, 15]. For example, the treatment dose regulated the number of responding cells in the population, exhibiting digital (binary) on-off activation [16–18], while the timing and amplitude of the NF-κB response controls target gene expression [11, 19].

Extreme temperature activates the Heat Shock (HS) response, causes protein denaturation and regulates many cellular processes. The HS response is mediated by a family of Heat Shock Factors (HSFs) and Heat Shock Proteins (HSPs) [2]. HSPs are the major molecular chaperones, which assist in protein folding, protect stress-labile proteins and contribute to proteolysis of damaged proteins. HSFs, on the other hand, act as TFs that regulate expression of HSPs, but also other proteins involved in the HS response. HS has been shown to modulate the NF-κB system [20–22]. High temperature inhibits cytokine-induced degradation of IκB, nuclear translocation of NF-κB and activation of NF-κB-dependent transcription [23–27]. This might involve attenuation of IKK activity due to temperature-mediated posttranslational changes leading to denaturation and loss of solubility [28, 29]. This is in part regulated via complex interactions with HSPs [30]. While HSPA1 [31, 32] and HSPB1 [33] bind and inhibit IKK activity, HSPA1 and HSP90 stabilize and facilitate renaturation of IKK [34, 35]. The action of HSPs in addition involves modulation of membrane receptor complexes upstream of IKK (including TRAF6 and TRAF2) [36, 37]. The attenuation of NF-κB activation could also depend on interactions between HSPA1 and NF-κB/IκBα (but not IKK/NEMO) [38], or with tertiary NF-κB/IκBα/IKK complexes [39]. It has been also reported that induction of HSPA1 expression by a mutated, constitutively active HSF1, in the absence of HS, did not suppress activation of the NF-κB pathway by TNFα [25]. Other potential mechanisms involve temperature-dependent changes in nuclear/cytoplasmic transport [40] as well as regulation of transcription and translation [41–43], which may affect many global cellular processes and in particular NF-κB signaling. Overall, current data suggest a multifaceted interaction between the NF-κB and the HS response systems. However, the precise molecular mechanisms of this cross-talk are not fully understood.

The temporal control of NF-κB activation by the coordinated degradation and synthesis of IκB proteins was first proposed by Hoffmann and co-workers [19]; [44]. A two-feedback model of the NF-κB regulatory module that included regulation of IKK and A20 was proposed by Lipniacki *et al*., 2004 [45], whose structure was subsequently extended [46–50]. Reduced versions of these models have also been developed and analyzed [51, 52]. The NF-κB response at the individual-cell level exhibits oscillatory dynamics and is subject to stochastic fluctuations [11], which renders oscillations in single-cells out of phase with each other in a population [47]. These stochastic dynamics have been extensively studied using single cell analysis and mathematical modeling, suggesting the involvement of both intrinsic and extrinsic noise [45–47, 53, 54]. Much less attention has been devoted to mathematical modeling of the HS response pathway, with existing work focusing on the intracellular processes that are initiated by the HS [55–57]. Recently, the work by Sheppard *et al*. demonstrated that the HS-inducible HSPA1 inhibits the population-level NF-κB system responses to TNFα simulation via number of different mechanisms, including the inhibition of IKK and the reduction of cellular p65 levels [32]. This provided important quantitative and mechanistic insights into NF-κB and HS crosstalk, but more studies are required to recapitulate the physiological context associated with elevated temperature, such as actions of different HSPs or stress-induced protein damage [24].

Here we used integrated modeling and experimental approaches to quantitatively characterize the crosstalk mechanisms involved in HS-regulated modulation of the NF-κB response in cellular populations and single cells. Using immunoblotting analysis we showed that the exposure to clinically relevant high temperature [58] resulted in time-dependent attenuation of TNFα-induced p65-Ser536 phosphorylation in populations of human osteosarcoma cells. In order to understand how this behavior is controlled in single cells, we used our mathematical model of the NF-κB signaling system [54] to systematically implement different putative mechanisms of temperature-dependent regulation. With use of time-lapse imaging of a NF-κB fluorescent reporter we observed that dampening of the population-level NF-κB responses involves “all-or-nothing” encoding at the single cell level. We found that while most of the cells did not show a NF-κB response to TNFα stimulation after 60 min HS, a small fraction gave a response similar to that of cells without HS. Furthermore, we show experimentally that HS stimulation was associated with decrease of cellular IKKα and IKKβ levels, but a combination of crosstalk mechanisms was required to recapitulate the single cell data. Overall, we demonstrate that following HS and stimulation the NF-κB system exhibits unintuitive responses, which demonstrate the need for single cell analyses to truly understand underlying mechanisms.

## Results

### Heat shock attenuates population-level NF-κB responses to TNFα stimulation

**Fig 1:**
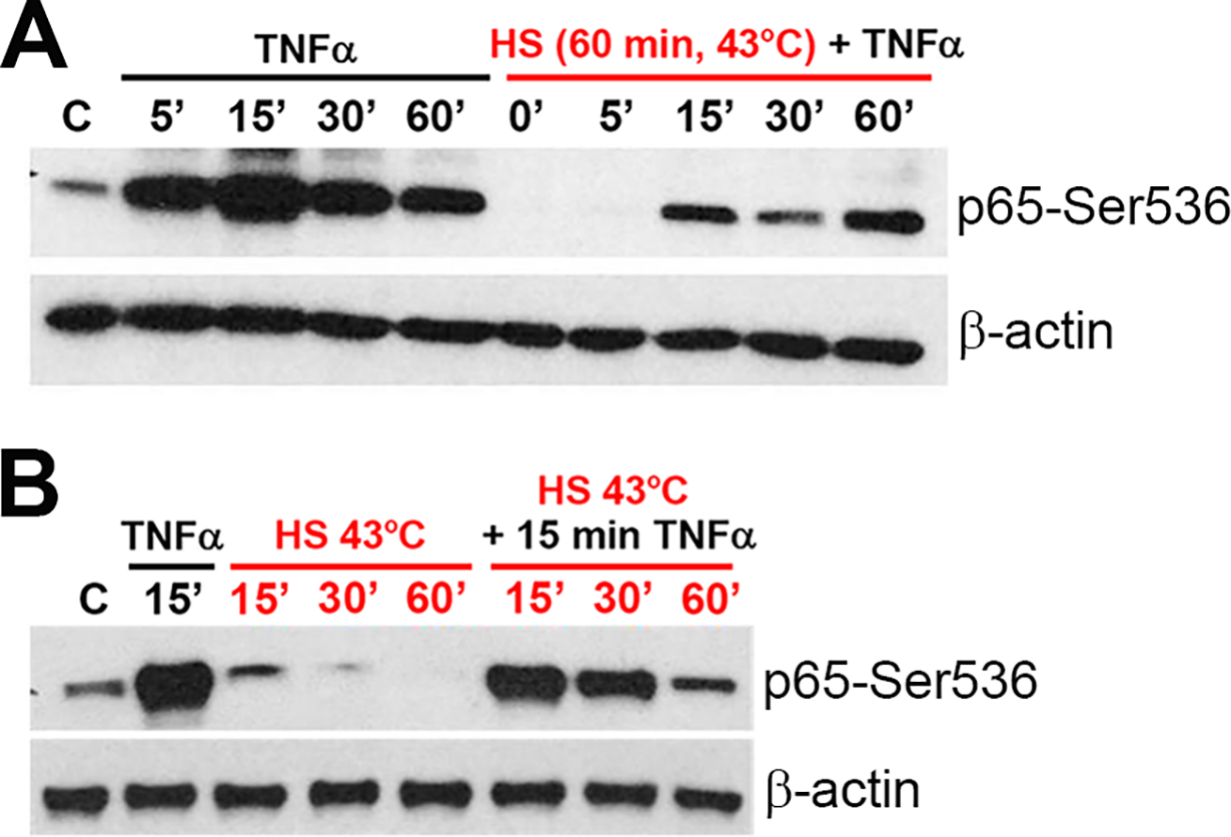
TNFα-induced p65-Ser536 phosphorylation is inhibited by prolonged HS. The level of p65-Ser536 phosphorylation was analyzed by Western blot in the whole U2OS cells lysates (representative example from three biological replicates). (**A**) Cells either cultured under normal conditions (37°C) or subjected to 60 min HS at 43°C were treated with TNFα for indicated times (min in black). (**B**) Cells were exposed to 43°C HS for indicated times (min in red) and subsequently treated with TNFα for 15 min. Shown also are appropriate controls (C denotes no HS no TNFα). β-actin expression was used as a loading control.

In order to characterize the HS-regulated NF-κB cell population response, we first utilized immunoblotting to measure Ser536 phosphorylation of the NF-κB p65 subunit as a proxy for TNFα-mediated NF-κB system activation. As expected [25–28], treatment of human osteosarcoma (U2OS) cells maintained at 37°C resulted in rapid and robust NF-κB activation (Fig 1A). As early as 5 min after TNFα addition there was increased Ser536 phosphorylation of NF-κB p65 subunit (in comparison to untreated cells), which peaked 15 min after treatment. In comparison, 60 min exposure to 43°C HS (see Materials and Methods) resulted in a substantial reduction in Ser536 phosphorylation. In these cells, the highest level p65-Ser536 phosphorylation was observed 60 min after TNFα treatment and at shorter times was much lower than in cells without HS. The attenuation of the NF-κB response depended on the duration of the HS exposure; while no change was observed after 15 min HS (in comparison to cells maintained at 37°C), substantial inhibition of p65-Ser536 phosphorylation was observed after a longer HS duration (Fig 1B). Overall, these data demonstrate that HS-exposure induced a substantial reduction of the population-level NF-κB activity and this effect depends on the HS exposure duration.

### Mathematical modeling discriminates heat shock mechanisms in single cells

**Fig 2:**
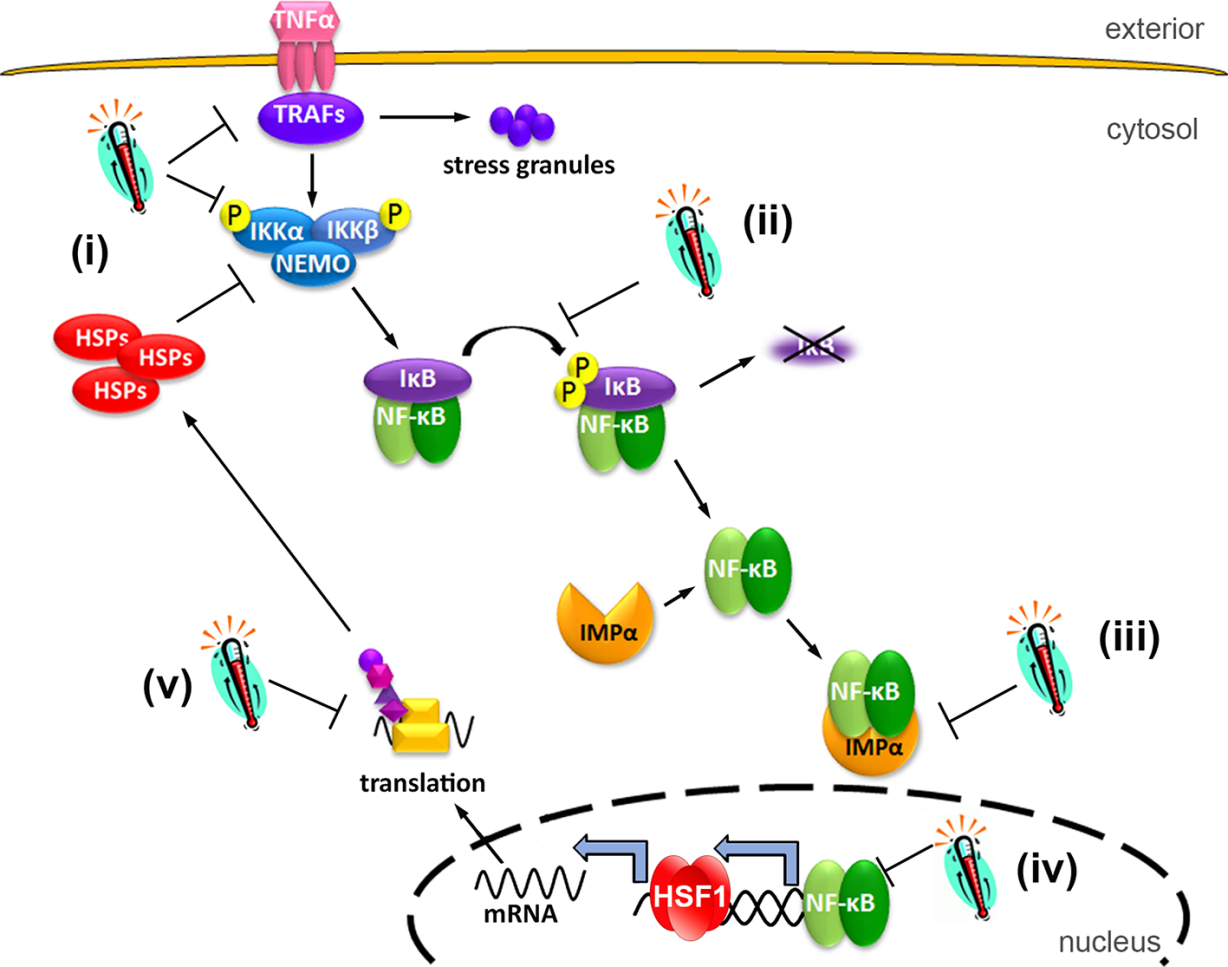
Schematic diagram of the HS-induced NF-κB regulation. HS may lead to: (i) IKK inactivation due to conformational changes or denaturation, or actions of HSPs and upstream kinases; (ii) inhibition of IκB phosphorylation; (iii) modulation of importin-dependent nuclear transport; (iv) destabilization of protein complexes involved in transcription; (v) inhibition of translation via molecular crowding and stress granules formation.

Current data suggest a multilayered interaction between the NF-κB and the HS response systems [32], but the precise molecular mechanisms are not fully understood. The previously hypothesized mechanisms involve HS-mediated modulation of the key processes in the NF-κB signaling network including (Fig 2):

(i) IKK activation; either due to competition for IKK among HS-induced proteins [31, 33] or to actions exerted upstream of IKK [59] or due to conformational changes of IKK proteins and their denaturation making them inaccessible to upstream kinases [34, 35];
(ii) IκBα phosphorylation by activated IKK [38, 39];
(iii) nuclear transport of NF-κB, due to changes in importin activities resulting from HS [40];
(iv) NF-κB-dependent transcription; due to decreased stability of large protein complexes at higher temperatures, or interference via HS-dependent regulation (e.g. HSF1) [41, 42];
(v) A20 and IκBα translation; due to the creation of stress granules [43] or molecular crowding resulting from overproduction of HSPs.

**Fig 3:**
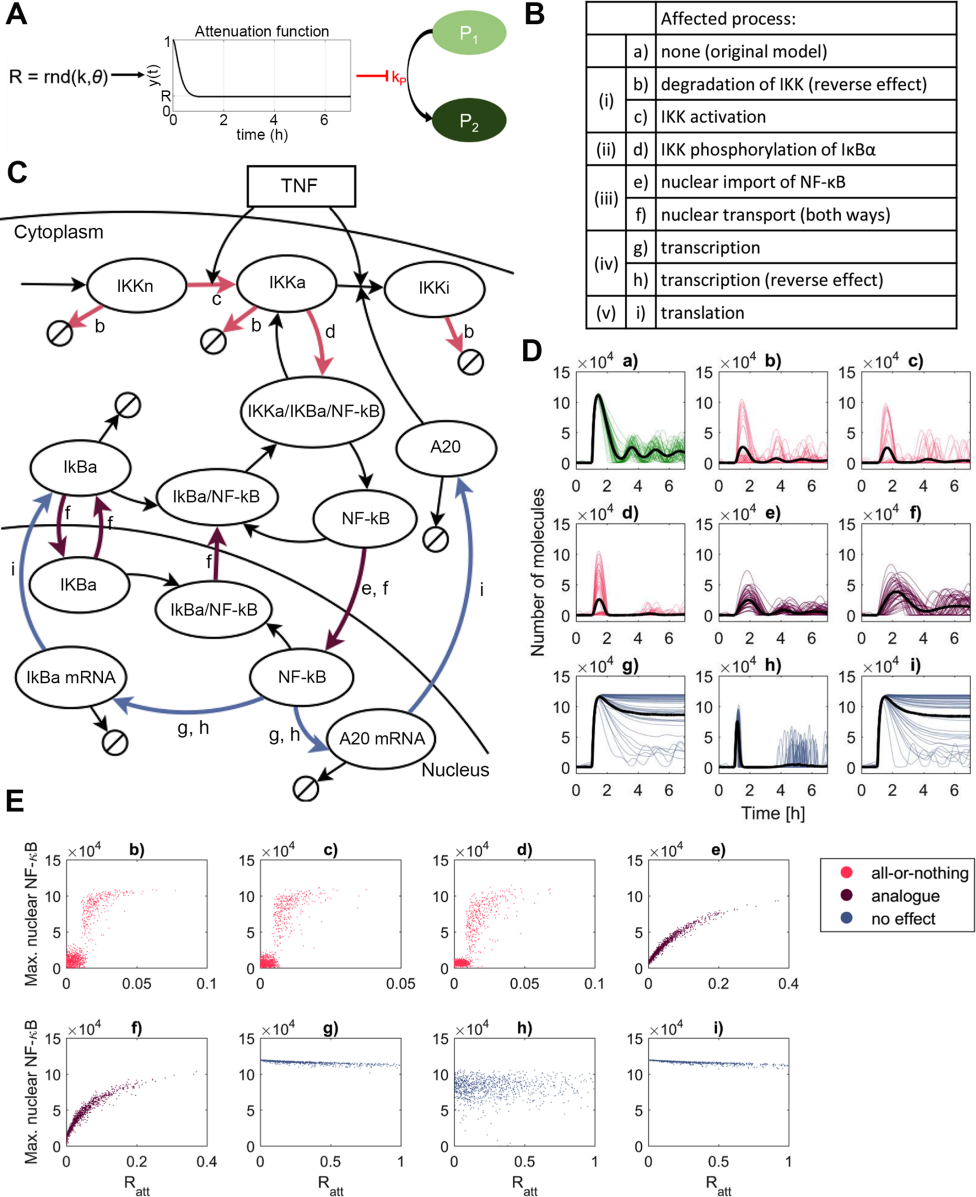
Mathematical modeling discriminates different single cell HS encoding mechanisms. (**A**) HS effect is modeled via a time-dependent attenuation function y(t). Each model simulation consists of three steps: (I) randomization of the attenuation coefficient *R* from the gamma distribution, (II) calculation of the attenuation function y(t) for the given *R*, (III) NF-κB model simulation. (**B**) List of considered cross-talk mechanisms. Roman numerals refer to mechanisms depicted in Fig 2. Reverse effect indicates activation. (**C**) Schematic diagram of NF-κB model. Colored arrows indicate simulated cross-talk mechanism from B. (**D**) Simulation of hypothetical mechanisms involved in the NF-κB and HS pathway cross-talk. Simulations performed for 60 min HS exposure before TNFα stimulation. Shown are a sample of 50 time courses of simulated nuclear NF-κB levels (colored lines) and average nuclear NF-κB levels (black bold line), calculated from 1,000 single cell simulations for cells treated with TNFα. (**E**) Scatterplots of the maximum nuclear NF-κB against the attenuation coefficient *R* per simulated cell. Color scheme refers to “all-or-nothing” or “analogue” encoding, as well as mechanisms where a population-level fit was not obtained (no effect).

In order to quantitatively understand how the experimentally observed HS-dependent inhibition of the NF-κB signaling is regulated we implemented and systematically tested several plausible mechanisms by using mathematical modeling. Previous analyses demonstrated that the NF-κB system behavior in individual cells may exhibit substantial heterogeneity [46, 47]. For example, low-dose TNFα stimulation may only activate a small fraction of cells in the population, resulting in “all-or-nothing” single cell encoding [16–18]. This means that the population averages may not only mask true single cell behavior, but also confound mechanistic studies. Therefore, in order to interpret observed population-level NF-κB responses in terms of the single cell behavior we used our previously developed stochastic model of the NF-κB system [54]. HS-induced crosstalk was incorporated in the NF-κB model via time-dependent attenuation function (Fig 3A; see also Eq (1) and the Mathematical Model section). This function was used to systematically modify the constant rate coefficients *k*_*i*_ associated with different processes, which were hypothesized in the model to be affected by the HS (Fig 3B and 3C, see also Table 1). To account for heterogeneity in the cellular sensitivity to HS, for each cell, the attenuation coefficient *R* describing the amplitude of the attenuation function has been sampled from a gamma distribution. The smaller the *R* values are, the greater are the changes of the corresponding rate parameter in the model and thus the stronger HS inhibition. The values of *R* (acting on different model parameters, respectively, Table 1) have been fitted (if possible) to obtain an 80% reduction of the population level nuclear NF-κB responses (estimated as an ensemble average of 1,000 simulated single cells, in comparison to control cells, Fig 3D).

**Table 1:** Shape (*k*) and scale (*θ*) parameters of gamma distribution for the attenuation coefficient. Note that for *k = 1*, θ corresponds to the average value of the attenuation coefficient *R*.

Mechanism considered		*y*_*i*_(*t*)	*k*	*θ*	Modified model parameter
A. One mechanism at a time (Fig 3)					
b	Degradation of IKK	*y*_*b*_(*t*)	1	0.01	*k*_*deg*_
c	Inhibition of IKK activation	*y*_*c*_(*t*)	1	0.0065	*k*_*1*_
d	Inhibition of IκBα phosphorylation	*y*_*d*_(*t*)	1	0.01	*a*_*3*_
e	Inhibition of nuclear import of NF-κB	*y*_*e*_(*t*)	1	0.05	*i*_*1*_
f	Inhibition of nuclear transport (both ways)	*y*_*f*_(*t*)	1	0.05	*i*_*1*_, *i*_*1a*_, *e*_*1a*_, *e*_*2a*_,
g	Inhibition of transcription	*y*_*g*_(*t*)	1	0.3	*c*_*1*_, *c*_*2*_, *c*_*1a*_, *c*_*2a*_
h	Induction of transcription	*y*_*h*_(*t*)	1	0.3	*c*_*1*_, *c*_*2*_, *c*_*1a*_, *c*_*2a*_
i	Inhibition of translation	*y*_*i*_(*t*)	1	0.3	*c*_*4*_, *c*_*4a*_
B. Multiple mechanisms contributing at the same time (Fig 7)					
b*+c	Degradation of IKK (constant) + inhibition of IKK activation	*y*_*c*_(*t*)	1	0.013	*k*_*1*_
b*+d	Degradation of IKK (constant) + inhibition of IκBα phosphorylation	*y*_*d*_(*t*)	1	0.018	*a*_*3*_
b*+e	Degradation of IKK (constant) + inhibition of nuclear import of NF-κB	*y*_*e*_(*t*)	1	0.075	*i*_*1*_
b*+c+e	Degradation of IKK (constant) + inhibition of IKK activation + inhibition of nuclear import of NF-κB	*y*_*c*_(*t*) *y*_*e*_(*t*)	1 1	0.026 0.18	*k*_*1*_ *i*_*1*_
b*+d+e	Degradation of IKK (constant) + inhibition of IκBα phosphorylation + inhibition of nuclear import of NF-κB	*y*_*d*_(*t*) *y*_*e*_(*t*)	1 1	0.036 0.3	*a*_*3*_ *i*_*1*_

Simulations of control cells (TNFα stimulation, no HS exposure, Fig 3D, mechanism a) show oscillatory NF-κB responses in individual cells, with a rapid first nuclear NF-κB translocation characterized by a high amplitude (up to 10^5^ NF-κB molecules). The first peak of the nuclear NF-κB translocation is homogeneous between individual cells, therefore the population average tightly recapitulates that of single cell responses. Over time, single cell and population-level responses diverge. This effect is due to variability incorporated in the model via: (1) distributed cellular IKK levels [16, 47] (sampled from a log-normal distribution, see Mathematical Modeling section), which indirectly regulate the timing and strength of the IκBα feedback; (2) molecular noise due to stochastic activation of IκBα and A20 feedback genes, as assumed in the model [54], which affects the amplitude and timing of the long-term NF-κB oscillations.

Simulation of HS exposure in the model confirmed that for most of the hypothesized mechanisms a substantial inhibition of the population-level NF-κB response can be achieved in the model (Fig 3D, mechanisms b-f). A notable exception was the modulation of IκBα/A20 feedback gene transcription and translation (Fig 3D, g-i, respectively). Inhibition resulted in a prolonged nuclear localization of the NF-κB, without affecting the timing and amplitude of the initial NF-κB translocation (in contrary to our experimental analyses). In contrast, up-regulation of feedback gene expression did not affect the amplitude of the first NF-κB translocation (Fig 3D, h). This confirmed that modulation of NF-κB feedback regulation is too slow to be a primary driver of the HS-induced response. Instead, the observed rapid inhibition of NF-κB signaling (within first 30 min after stimulation) might be associated with modulation of the TNFα binding, signal transduction to IKK, IKK activation and subsequently IκBα degradation and nuclear NF-κB transport [53]. In agreement, simulation of these crosstalk mechanisms shows a considerably damped population-level NF-κB response, consistent with experimental analyses (Fig 3C, b-f). Importantly, we found that the simulated single cell NF-κB trajectories allow discrimination of different modes of HS-mediated regulation. In particular, the modulation of the IKK activity and IκBα phosphorylation (Fig 3C, b-d), resulted in “all-or-nothing” NF-κB responses at the single cell level; while most of the cells did not show any considerable activation, a small fraction of cells exhibited NF-κB responses similar to those of control cells. In this case the relationship between the attenuation coefficient *R* and peak nuclear NF-κB amplitude reveals a clear transition between the responding and non-responding population (Fig 3D, b-d) resulting in a bimodal nuclear NF-κB distribution (S1 Fig). This transition is sharper for the mechanisms involving IKK inhibition in comparison to that of IκBα phosphorylation, and independent of the IKK level (S1A Fig). In contrast to IKK related-parameters, modulation of the parameters associated with nuclear/cytoplasmic NF-κB transport involves “analogue” encoding; the peak nuclear amplitude was tightly correlated with *R*, resulting in HS-mediated inhibition of all cells and unimodal distribution of the peak nuclear NF-κB in the population (Figs 3D and 3E, e-f, and S1).

Overall these simulations demonstrate that a mathematical model of the NF-κB system may discriminate between different HS crosstalk mechanisms and suggests that single cell approaches are required to accurately interpret population-level experimental data.

### Single cell imaging reveals “all-or-nothing” encoding of NF-κB responses upon HS exposure

**Fig 4:**
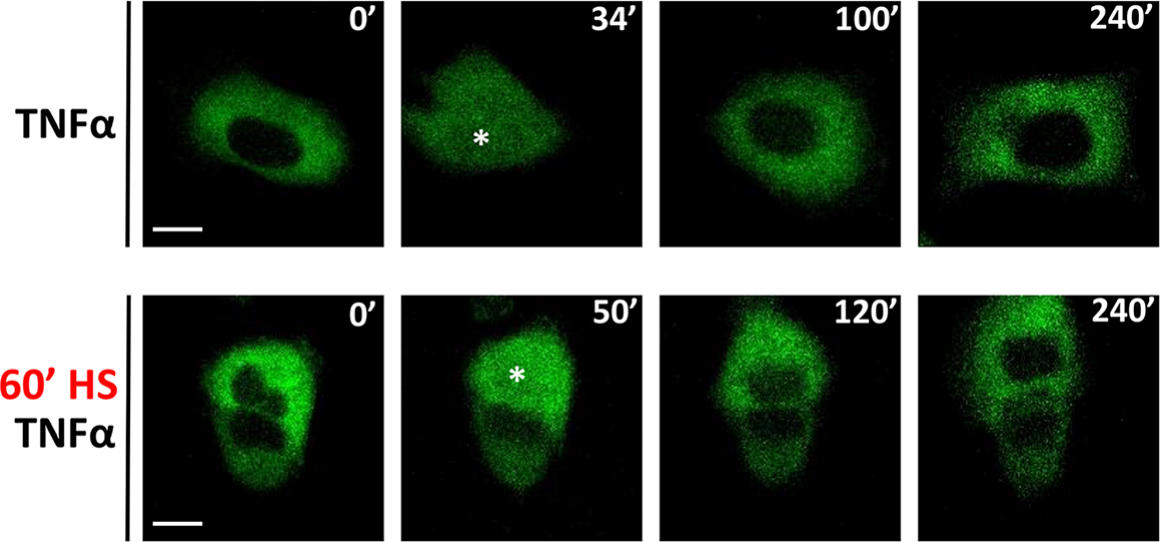
HS inhibits single cell p65-EGFP translocation. Confocal microscopy images of representative U2OS cells stably expressing p65-EGFP fusion protein. Top panel: cells maintained at 37°C and stimulated with TNFα. Bottom panel: cells exposed to 60 min 43°C HS prior to TNFα stimulation. Time of stimulation is displayed in minutes. Nuclear p65-EGFP translocation is depicted with an asterisk (*). Scale bar, 10 μm.

**Fig 5:**
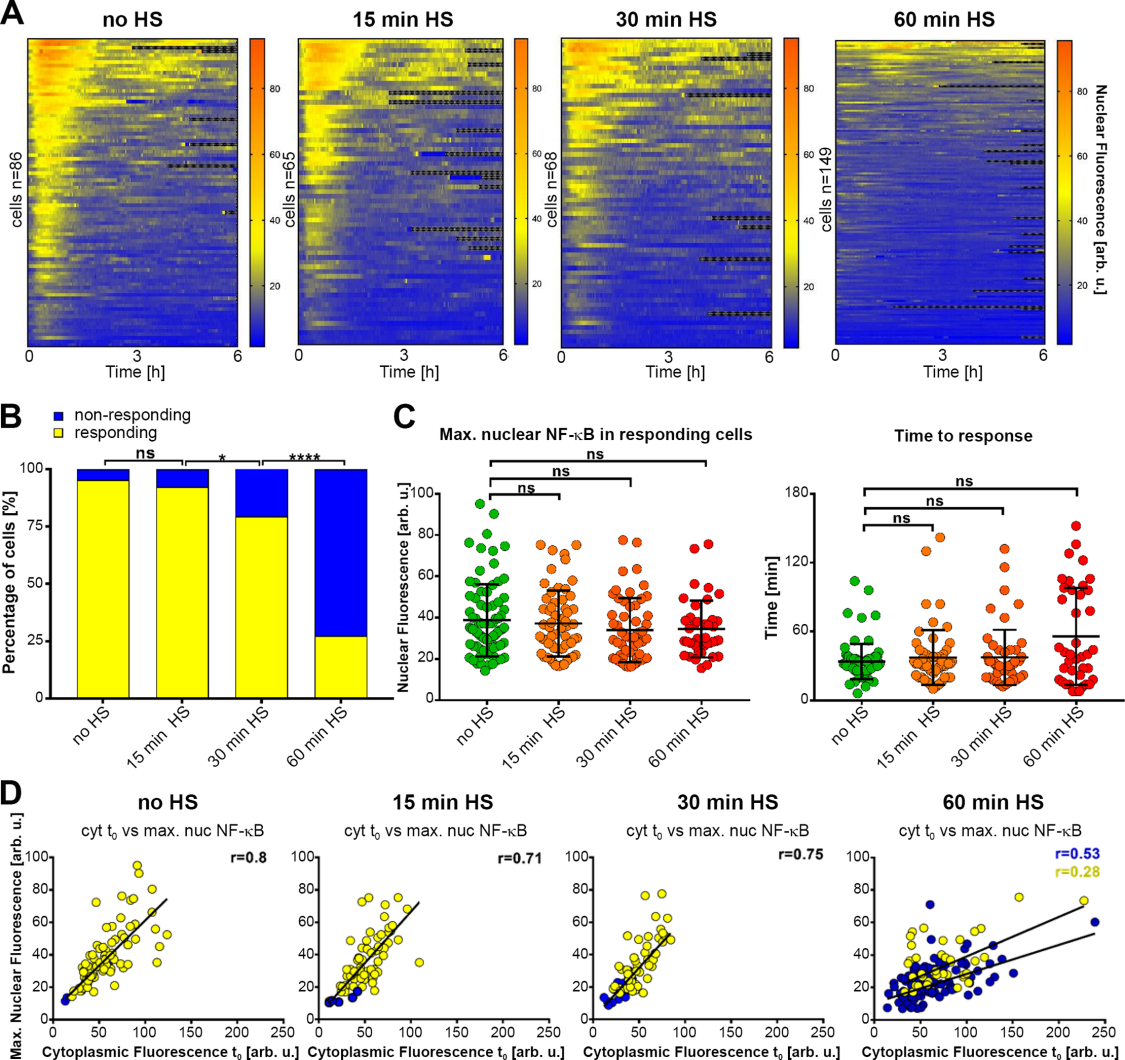
HS can alter single cell NF-κB responses to TNFα stimulation. (**A**) Heat maps of clustered nuclear NF-κB trajectories in U2OS cells stably expressing p65-EGFP fusion protein. Cells exposed to 43°C HS for indicated times and treated with TNFα. n = 86, 65, 68, 146 indicates number of cells imaged and analyzed. (**B**) Percentage (%) of responding (yellow) and non-responding (blue) cells to TNFα, maintained at 37°C or exposed to HS prior to treatment (for data in A). Statistical difference assessed with Chi-square test (*P value < 0.01, ****P value < 0.0001, ns- not significant). (**C**) Characteristics of single cell NF-κB responses. Analysis of the single cell traces of responding cells from A. Left panel: the distribution of the maximum nuclear p65-EGFP. Right panel: time to first response. Individual cell data depicted with circles (with mean ± SD per condition). Kruskal-Wallis one-way ANOVA with Dunn’s multiple comparisons test was used (ns–not significant). (**D**) Correlation between cytoplasmic fluorescence at time t_0_ and maximal nuclear fluorescence for cells cultured in normal conditions or subjected to 15, 30 and 60 min of HS (for data in A). Responding cells depicted with yellow circles, non-responding with blue, with fitted regression lines and Spearman correlation coefficient (r), respectively.

In order to monitor the HS-mediated inhibition of the NF-κB system at the single cell level, we used retroviral transduction to produce a U2OS line stably expressing fluorescently-labeled NF-κB p65 (p65-EGFP) [11]. In agreement with analyses of wild-type cells, transduced cells exhibited HS-mediated attenuation of NF-κB p65-Ser536 phosphorylation at the population-level with similar kinetics for both the fluorescently tagged and native protein (S2 Fig). Using confocal microscopy we assayed TNFα-induced NF-κB responses in single cells under HS exposure conditions ranging from 15 to 60 min (see Fig 4 for confocal images of representative cells, and Figs 5 and S3 for analyses of single cell traces). 95% (82 of 86) of TNFα treated cells which were not exposed to temperature (no HS control) exhibited a rapid nuclear p65-EGFP translocation within minutes, in agreement with p65-Ser536 phosphorylation analyses (Fig 5A and 5B). The amplitude of the initial NF-κB translocation was variable among individual cells (Fig 5C). Subsequent NF-κB translocations were also observed, with up to three per individual cell within a 6-hour window (S3A Fig). These patterns however were less robust in comparison to previously described single cell NF-κB oscillations in different cell types [11, 46, 53]. In agreement with the immunoblotting analyses, imaging data across nearly 300 cells demonstrated inhibition of NF-κB signaling following HS exposure (Figs 5A and S3A). 92% (60 of 65) of cells exhibited a clear NF-κB nuclear translocation in response to TNFα treatment after 15 min HS exposure, however significantly fewer cells (79%, 54 of 68) responded after 30 min HS exposure. After 60 min HS exposure 27% (40 of 146) of cells exhibited NF-κB translocations following TNFα treatment. Importantly, there was no statistical difference in the peak amplitude or timing of the nuclear NF-κB p65 in responding cells across all conditions (in comparison to no HS treatment, Fig 5C).

The single cell data demonstrated that the amplitude of the nuclear NF-κB responses substantially varied across individual cells (Fig 5A). However, we found that the level of the NF-κB response per cell (i.e. the amplitude of the nuclear translocation) was directly proportional to the total cellular NF-κB level. We found that in unstimulated cells (at t = 0 min) there was an equilibrium between the cytoplasmic and nuclear NF-κB p65-EGFP across all single-cell data (as shown via high Spearman correlation coefficient r>0.75, S3B Fig). Similar high correlations were found between pre-stimulation nuclear (or cytoplasmic) and the TNFα-induced nuclear NF-κB p65-EGFP translocation amplitude (Figs 5D and S3B). However, the analysis of different translocation patterns demonstrated that whether the cell responded or not, was not dependent on the cellular level of p65-EGFP (Fig 5D).

Overall our time-lapse imaging data show that HS crosstalk is consistent with “all-of-nothing” single cell encoding. At longer HS exposure times fewer cells exhibited NF-κB activation following TNFα treatment (Fig 5B). However, the cells that did respond to TNFα showed similar NF-κB kinetics to those without HS (Fig 5C). Importantly, this behavior was preserved in those data normalized in terms of the net nuclear translocation [60] (S3C Fig).

### HS-dependent depletion of IKK is not sufficient to recapitulate observed responses

**Fig 6:**
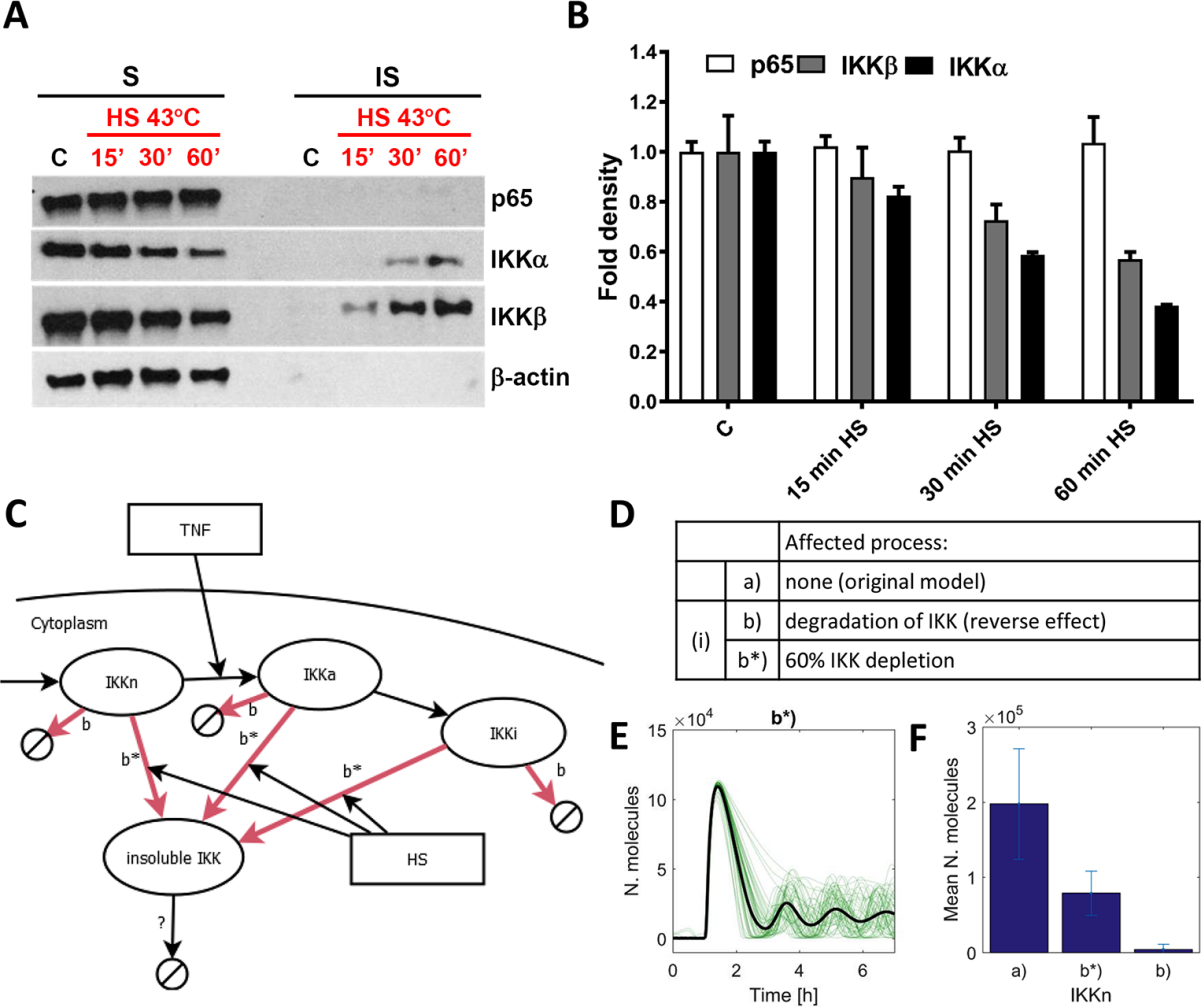
IKK depletion was not sufficient to recapitulate observed responses. **(A)** Western blot analysis of soluble (S) and insoluble (IS) NF-κB p65, IKKα and IKKβ proteins level in WT U2OS cells. Cells were either cultured under normal conditions (37°C) or subjected to 15, 30 and 60 min of HS at 43°C. β-actin was used as a loading control. (**B**) Quantification of the blotting data from A. (**C**) Schematic representation of the IKK modulation in the model. (**D**) List of considered models (as indicated with red arrows in schematics C). (**E**) Simulation of the HS-dependent 60% IKK depletion (mechanism b*). Shown are 50 time courses of simulated nuclear NF-κB levels (green lines) and average nuclear NF-κB levels (black bold line), calculated from 1,000 single cell simulations for cells treated with TNFα after 60 min HS exposure. **(F)** Comparison of the cellular IKK levels after 60 min HS exposure. Shown are mean (±SD) levels for models assuming no inhibition (a, as in Fig 3), as well as HS-mediated IKK depletion (mechanism b*) and IKK degradation (b, as in Fig 3).

The experimentally observed single cell responses are consisted with “all-or-nothing” encoding mechanisms mediated via the inhibition of IKK activity or IκBα phosphorylation, but not the “analogue” modulation of the NF-κB import/export (see Fig 3 for mechanisms b-d and e-f, respectively). We therefore sought to validate this prediction experimentally. Many potential mechanisms have been suggested previously, including interactions between HSPs and IKK [30–35] or membrane receptor complexes upstream of IKK [36, 37] as well as NF-κB/IκBα [38, 39]. In order to explain the complete lack of signaling responses in a fraction of cells, a rapid and complete change of the nature of these interactions is required upon HS exposure. A second line of evidence suggests that HS might attenuate IKK kinase activity by temperature-mediated posttranslational changes leading to loss of its solubility, limiting the amount of IKK, which can be activated [28, 29]. We therefore measured the levels of soluble and insoluble IKKα and IKKβ subunits responsible for kinase activity [3] in cells exposed to HS for different durations of time (Fig 6A). In agreement with our single cell and population level data on NF-κB activity (Figs 1 and 5), we found HS time-dependent changes in soluble and insoluble IKKα and IKKβ in whole U2OS cell lysates (Fig 6A). The level of IKKα and β was reduced to 40 and 60%, respectively after 1h HS treatment (60 and 70% after 30 min), but not after 15 min treatment as assessed following quantification of the immunoblotting data (Fig 6B). The time-dependent loss of soluble IKK form was mirrored by the increases of insoluble IKK levels which was particularly apparent for IKKβ after 60 min HS exposure. In contrast, the levels of soluble (and insoluble) NF-κB p65 or the β-actin loading control remained unaffected by HS exposure. Altogether these data suggest a mechanism where HS-exposure reduced the effective level of cellular IKK, which could subsequently be activated by the cytokine treatment. We used mathematical modeling to investigate this mechanisms in detail. We assumed a distribution of cellular IKK levels across population (as in Fig 3) and that all forms of the IKK were removed from the system by a common HS-mediated process (and common degradation rate equally affecting all cells, model b*, Fig 6C, 6D and 6E). In this model, the 60% depletion was not sufficient to obtain the appropriate fraction of responding cells and their amplitude (Fig 6E and 6F). We found that this required 98% depletion of IKK in the model, suggesting that other cross-talk mechanisms might be involved.

### Predicted HS crosstalk involves co-operation between multiple “all-or-nothing” mechanisms

**Fig 7:**
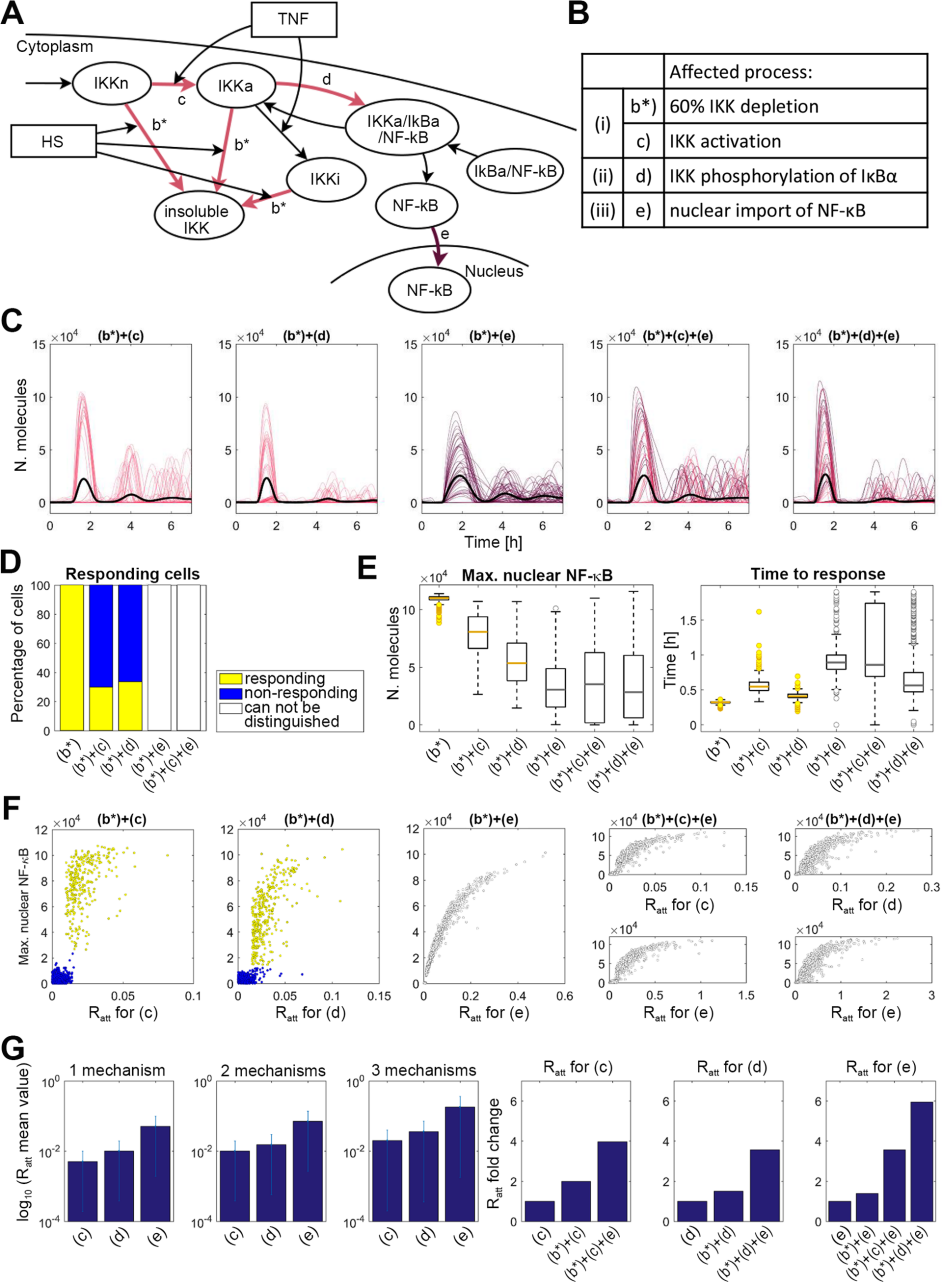
HS cross-talk involves multiple “all-or-nothing” mechanisms. (**A**) Schematic representation of the IKK modulation schemes in the model. (**B**) List of considered cross-talk mechanisms (as indicated with colored arrows in A). (**C**) Simulation of HS cross-talk mechanisms. All models assume HS-mediated 60% depletion of IKK (mechanism b*) in combination with other mechanisms as in B. Shown are a sample of 50 time courses of simulated nuclear NF-κB levels (colored lines) and average nuclear NF-κB levels (black bold line), calculated from 1,000 single cell simulations for cells treated with TNFα after 60 min HS exposure. **(D)** Percentage (%) of responding (yellow) and non-responding (blue) cells from data in C or Fig 6E (model b*). **(E)** Characteristics of NF-κB responses in cells from C. Left panel: the distribution of the maximum nuclear NF-κB. Right panel: time to first response. Shown are characteristics of responding cells for (b*+c) and (b*+d), and of all simulated cells for other mechanisms. (**F**) Scatterplots of the maximum nuclear NF-κB level against the attenuation coefficient *R* per cell for different models considered as in C. **(G)** Analysis of the HS-mediated inhibition across different mechanisms. (Left) Shown are the average (±SD) values of the mechanism-specific attenuation coefficient *R* across models in C. (Right): Fold change of the mechanism specific attenuation coefficient *R* across different models (calculated with respect to the corresponding *R* for a model without IKK depletion).

In order to investigate the likelihood of whether other cross-talk mechanisms might be involved [32], and how they might co-operate, we conducted a simulation study (Fig 7A and 7B). The 60% depletion of the otherwise distributed IKK level per cell (model b*) was fixed and combined with other mechanisms: model c for inhibition of IKK activation; model d for IκB phosphorylation; model e for nuclear import of NF-κB. Models incorporating combinations of different mechanisms (in twos and threes) were fitted to obtain 80% inhibition of the population-level NF-κB response after 60 min HS exposure (Fig 7C). The models were then assayed for their behavior at the single cell level (such as the fraction of responding cells, peak nuclear NF-κB amplitude and time to response, Fig 7D–7F).

We found that the combination of two “all-or-nothing” mechanisms (depletion of IKK with either inhibition of IKK activation or IκB phosphorylation) resulted in “all-or-nothing” responses (models b*+c or b*+d), which were able to recapitulate both the single cell and the population-level data (Fig 7C–7F). We found that these models were characterized by non-additive changes in the respective HS cross-talk inhibition. That is the 60% depletion of IKK, although not sufficient alone to reproduce the data, resulted in the >2-fold increase of the attenuation coefficient values (and thus lower inhibition) required to fit the data (e.g. comparing *R* for models b*+c vs. mechanism c alone, Fig 7G and Table 1 for parameter values). This suggests a model where a partial (rather than a complete) inhibition of multiple mechanisms of NF-κB signaling might allow robust encoding of HS responses.

We then asked what were the effects of combining mechanisms involving “analogue” encoding. We found that consideration of the HS-mediated NF-κB transport in addition to 60% IKK depletion (model b*+e) resulted in the analogue encoding of single cell responses, not consistent with experimental data (Fig 7D–7F). In addition, we also considered three mechanisms acting simultaneously, where HS-mediated inhibition of NF-κB transport was combined with IKK depletion and IKK activation or IκB phosphorylation (models b*+c+e and b*+d+e, respectively). We assumed that in individual cells, the respective attenuation coefficient values were correlated (i.e. HS simultaneously inhibited all mechanisms–see Materials and Methods). Based on the results of previous simulations, we assumed a 2-fold decrease in the inhibition of the IKK activation or IκB phosphorylation (compared to the model b*+c and b*+d), while the attenuation coefficients for the inhibition of NF-κB transport (mechanism e) were fitted (see Fig 7G and Table 1 for parameter values). Model simulations showed that in this case, the resulting single cell responses also exhibited “analogue encoding” not consistent with the data (Fig 7F).

**Fig 8:**
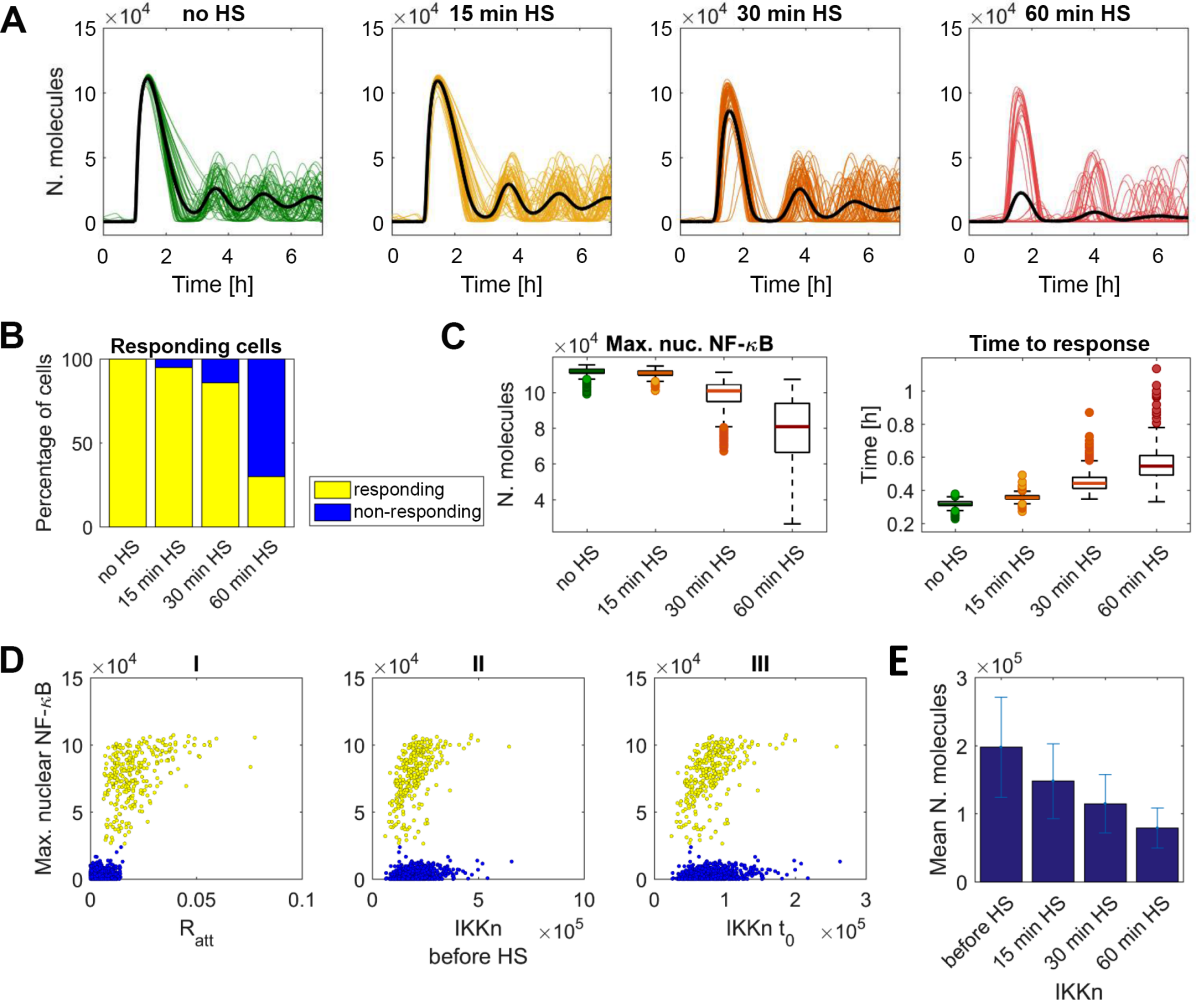
Temporal encoding of HS crosstalk. (**A**) Simulation of HS cross-talk assuming IKK depletion and inhibition of IKK activation (model b*+c from Fig 7). Shown are a sample of 50 time courses of simulated nuclear NF-κB levels (colored lines) and average nuclear NF-κB levels (black bold line), calculated from 1,000 single cell simulations for cells treated with TNFα after different HS exposure times. **(B)** Percentage (%) of responding (yellow) and non-responding (blue) cells from A. **(C)** Characteristics of NF-κB trajectories in responding cells from B. Left panel: the distribution of the maximum nuclear NF-κB. Right panel: time to first response. **(D)** Scatterplots of the maximum nuclear NF-κB level per cell against (I) attenuation coefficient *R*, (II) IKKn level before HS and (III) IKKn at time t_0_ (after 60 min HS, before TNFα treatment). **(E)** Fitted IKKn levels after different HS exposure times (calculated from data in A).

Therefore, these simulations suggested that combination of HS cross-talk mechanisms does involve predominantly IKK-mediated “all-or-nothing” encoding mechanisms, rather than mechanisms involving analogue encoding. In agreement, models assuming combination of IKK depletion and inhibition of IKK activation, were able to reproduce experimentally observed temporal HS-mediated NF-κB responses (Fig 8, also assuming random cellular distribution of the NF-κB levels; S4 Fig).

## Discussion

Temperature modulates many cellular processes, including the responses of the NF-κB signaling system [1]. Previous work suggested that the exposure to high temperature attenuates the NF-κB system responses and proposed several mechanisms involved in this process [23–27, 42]. Unfortunately, the quantitative understanding of these crosstalk processes is limited. Here, in order to distinguish between different hypotheses of crosstalk mechanisms, we performed a series of biological and *in silico* experiments assaying TNFα-induced NF-κB system responses in cells exposed to high 43°C temperature.

We designed our experiments such that HS exposure was transient, (for up to 1h), and immediately before TNFα stimulation at 37°C in order to limit potentially confounding systemic changes associated with long-term high temperature exposure or any delayed feedback via HSF regulation [21]. In agreement with the previous work [25–28], we found that the exposure to clinically relevant high temperature suppressed the population-level NF-κB system responses to TNFα stimulation in human osteosarcoma cells (Fig 1). As revealed by live-cell time-lapse imaging analyses of clonal NF-κB reporter cells, population-level inhibition was a result of digital “all-or-nothing” encoding of single cell response (Fig 3). That is, while most of the cells do not respond to TNFα stimulation after a 60 min HS exposure, approximately 27% had a response similar to that of the control cells. Shorter HS times resulted in larger numbers of responding cells as 79% of cells responded after 30 min HS, while 15 min HS was not statistically different from the control (Fig 3B). This behavior was similar to the previously observed digital activation in response to low dose stimulation [16–18] or repeated TNFα pulses [53], with the fraction of responding cells being proportional to the dose of stimulation (or pulsing intervals). Here the HS duration appears to differentially desensitize cells to TNFα stimulation. This study to our knowledge is the first to use live-cell time-lapse microscopy to study HS mediated NF-κB crosstalk. It indicates that the cellular HS pathways encode complex and unintuitive single cell response patterns.

Mathematical modeling allows improved quantitative and mechanistic understanding from experimental observations. The most up-to-date quantitative study of HS-related and NF-κB crosstalk was described by Sheppard *et al*. in order to understand the TNFα-induced NF-κB response in cell lines engineered to express different amounts of HS-inducible HSPA1 (otherwise known as HSP72) [32]. It was found that at the population-level HSPA1 overexpression reduced the amount of the cellular NF-κB as well as its DNA binding, IKK activity and IκBα phosphorylation usually seen following TNFα treatment. Mathematical modeling predicted an involvement of IKK signaling in the HSP crosstalk as a part of multi-faceted interaction involving in part reduction of the NF-κB p65 level and modulation of IκBα transcription. Using a similar computational methodology, here we used mathematical modeling to investigate the mechanisms involved in the HS and NF-κB crosstalk upon temperature exposure. We assumed one directional interaction where the HS effect was incorporated in the NF-κB system by modifying key model parameters in a time of exposure-dependent manner (Table 1). In agreement with the literature, we implemented several mechanisms of HS-mediated inhibition involving inhibition of IKK activation [31–35, 59], IκBα phosphorylation [38, 39], nuclear transport of the NF-κB [40] as well as inhibition of the NF-κB-dependent transcription [41, 42] and translation [43] of feedback genes (Fig 2). Critically, we used a single cell stochastic model of the NF-κB system [54] in order to interpret observed population-level NF-κB responses in terms of the single cell behavior and cellular heterogeneity [46, 47]. This approach showed that different NF-κB system parameters (encoding HS-mediated crosstalk) predict differential encoding of the HS-responses in single cells (Fig 3). That is, model simulations predict that inhibition of IKK activity and IκBα phosphorylation might result in”all-or-nothing” encoding resulting in a fraction of responding cells in the population, with an amplitude similar to that of the control TNFα stimulated cells (Fig 3). In contrast, parameters assuming modulation of NF-κB transport resulted in “analogue” encoding, where all cells are proportionally affected by HS exposure. Moreover, we also suggest that the combination of “analogue” encoding mechanisms with “all-or-nothing” mechanisms might also result in “analogue” single cell responses (Fig 7). While, both consistent with the population-level data, only the modulation of IKK activity (and IκBα phosphorylation, or combinations of, Figs 5 and 8) recapitulates our single-cell imaging analyses. Overall, the NF-κB system behavior in individual cells may exhibit substantial heterogeneity in different contexts [46, 47], and our analyses suggest that population averages may not only mask true single cell behavior, but also confound mechanistic interpretation.

The observed heterogeneity of single cell responses seen by microscopy was recapitulated by modeling using a variable HS effect in individual cells, characterized by the attenuation coefficient *R*, which was assumed to be distributed in a population of cells. This suggests that individual cells might differentially encode HS stimulation, due to cell-intrinsic parameters involved in the crosstalk with HS response pathways. While simplified in the mathematical model, these parameters might involve distinct (although correlated) processes. For example, inhibition of IKK activation has been known to involve actions of HSP70 and HSP90, which differentially regulate IKK kinase activity, either directly [31–33] or indirectly, by modulating the signal transduction pathway, upstream of IKK (e.g. via adapter molecules such as TRAFs [59]). The extreme but physiological temperature has been known to induce conformational changes of IKK proteins or their denaturation, which decreases the pool of IKK that can be activated [28]. Indeed we demonstrated a time-depended depletion of the soluble IKKα and IKKβ subunits, which are required for IKK activity [28] upon HS exposure (Fig 6). These were correlated with the changes of the NF-κB signaling responses to TNFα (Figs 1 and 5). In contrast, the cellular levels of soluble NF-κB p65 were not affected by temperature treatment. While the reduced expression of NF-κB p65 was previously shown in cells stably overexpressing HSPA1 in comparison to wild type cells [32], our analyses suggest that this effect might not be critical in acute responses to HS. Therefore, we did not consider this mechanism in our modelling analyses. However, we demonstrated via modeling that changes in IKK levels alone might not explain the observed HS-modulation at the single cell levels, however, we have shown that other regulatory processes also contribute to the observed behavior as suggested previously [32]. Interestingly, the effect of combining multiple mechanisms in the model is non-additive; it thus allows achieving robust encoding of HS responses via partial inhibition (Fig 7). It will be important in the future to understand how these specific HSP mechanisms are mediated and how the HSF feedback [21] regulates these processes.

Hyperthermia has been considered a promising strategy to sensitize cancer cells to treatment [58], with common therapies targeting NF-κB signaling pathway (e.g. via the use of clinical IKK inhibitors [61]). Our data show that although exposure to clinically relevant high temperatures attenuates NF-κB system responses, the underlying single cell behavior is surprisingly complex and unintuitive. This suggests that effects of the treatment may not be easily predictable without a quantitative understanding of the mechanisms involved in the interactions between HS and NF-κB signaling. That is, the desired effect such as attenuation of the NF-κB activity, and subsequently cancer cell death might require a precise understanding of the exposure time and specific temperatures, or in fact other related parameters [58], in a multicellular tumor environment. It is particularly important to understand if the observed single cell responses are intrinsically stochastic, or imprinted, or in fact cytokine-dependent. This range of possibilities involves some cells being programmed to exhibit specific NF-κB system responses to cytokine stimulation [53]. Some cells could potentially exhibit resistance even after long exposure times, affecting treatment strategies [58]. Further studies are required to understand how the observed cellular heterogeneity can be controlled or in fact removed, in order to increase the overall sensitivity to treatment.

## Materials and methods

### Cell culture and reagents

Experiments were performed using the human U2OS osteosarcoma cell line (purchased from ATCC; cat. no. HTB-96). Cells were cultured at 37°C in humidified 5% CO_2_ in DMEM High Glucose medium (Sigma-Aldrich) supplemented with 10% (v/v) heat-inactivated fetal calf serum and routinely tested for mycoplasma contamination. For Western blotting, HS response was induced by transferring cells into a water bath at 43°C. After indicated HS times, cells were supplemented with fresh 37°C media. For confocal microscopy experiments, cells were transferred between incubators set at 43°C and 37°C, cells were supplied with fresh media at the target temperature before every transfer. 10 ng/ml of human recombinant TNFα (Sigma-Aldrich) was used to simulate NF-κB responses for the indicated time lengths.

### Engineering of p65-EGFP stably transfected U2OS cell line

p65-EGFP sequence was re-cloned from p65-EGFP-N1 plasmid [11] into pLNCX2 vector (Clontech) for expression under CMV promoter (using *HindIII* and *Not1* restriction sites). The Retroviral Gene Transfer protocol (Clontech) was followed for to obtain stable U2OS line expressing the resulting p65-EGFP-pLNCX2 vector. In brief, RetroPack PT67 packing cells were transfected with p65-EGFP-pLNCX-2 vector using TurboFect (Thermo Scientific), then cells were selected with G418 geneticin sulphate (Gibco), and the virus-containing medium was collected after one week of culture. U2OS cells were exposed to the virus-containing medium and stably transduced cells were selected with G418 (1.25 mg/ml) for one week.

### Protein extraction and western blotting

Cells were lysed in 1% NP-40, 0.5% sodium deoxycholate and 0.1% SDS in PBS, supplemented with Complete (Roche) protease and phosphatase inhibitor cocktail, centrifuged for 20 min at 14,000 rpm at 4°C. The supernatants, defined as a soluble fraction, were collected. Insoluble proteins, remaining in the pellets, were dissolved in a SDS sample buffer consisting of 25 mM Tris-HCl pH 6.8, 0.5% SDS, 2.5% glycerol, and 15% 2-mercaptoethanol. Protein concentration was determined by BCA assay (Thermo Scientific). Samples and ladder (Bio-Rad, #161–0375) were resolved on polyacrylamide gels and transferred to nitrocellulose membranes (Amersham), incubated 1 h at room temperature in blocking buffer (5% (w/v) non-fat milk powder in TBS-T containing 0.25 M Tris–HCl pH 7.5, 0.1% Tween-20, 0.15 M NaCl), washed 3 times in TBS-T and incubated overnight with primary antibody (p65-S536, CST #3033; IKKα, CST #11930S; IKKβ, CST #2684S; β-actin, Sigma-Aldrich A3854) at 1:1,000 dilution in blocking buffer. Membranes were washed 3 times in TBS-T and incubated with 1:1,000 HRP-conjugated secondary antibody for 1 h at RT. Membranes were washed (3 x TBS-T) then incubated with Luminata Crescendo Western HRP Substrate (EMD Millipore Corp.) and the signal was detected by exposure to Carestream Kodak BioMax MR film (Sigma-Aldrich). Densitometry analysis of IKKα, IKKβ and p65 bands were performed in ImageJ (in technical triplicates), quantified expression levels were displayed as a fold change vs. untreated condition.

### Confocal microscopy

Cells were plated onto 35 mm-glass-bottomed dishes (Greiner Bio-One) one day prior to the experiment and incubated on the microscope stage at 37°C in humidified 5% CO_2_. Two Carl Zeiss confocal microscopes were used (LSM710, AxioObserver and LSM780 AxioObserver) with Fluar 40x/1.30 NA Oil objectives. The 488 nm (ATOF set at 4%) line from an argon ion laser was used to excite the p65-EGFP fusion protein and emitted light between 490–540 nm was detected through pinholes set to 5μm. Image capture was performed using the Zeiss Zen 2010b software. Quantification of p65-EGFP nuclear fluorescence (or cytoplasmic fluorescence at t0) was performed using Cell Tracker (version 0.6) using region of interest analysis [62]. The data was exported as mean fluorescence intensity. TNFα-induced nuclear NF-κB responses in U2OS cells were less robust in comparison to previously described single cell oscillations in different cell types [11, 46, 53]. In particular, the first peak translocation had a relatively low amplitude (Fig 4). A cell was classified to have responded if >10% change in the nuclear NF-κB p65-EGFP level (in comparison to a basal unstimulated level at t = 0) was observed for >6 consecutive imaging time-points (equivalent to >12 mins).

### Mathematical modeling

The mathematical model considered the one-directional effects of HS on the NF-κB system. We used our two-feedback stochastic model of NF-κB signaling [54], which was subsequently modified to take into account actions exerted by the potential HS-induced processes. The modifications involve introducing HS-dependent temporal changes into specific rate coefficients of the original model (Fig 3). This line of reasoning is similar to the one presented in [32]. Each mechanism has been tested separately, by numerical simulation of NF-κB system response measured by changes in nuclear NF-κB level. By analyzing the experimental data (Fig 1), we noticed that the relationship between the duration of the heat shock and the level of the NF-κB system attenuation is nonlinear. This relationship follows a sigmoid shape similar to repressor Hill function, however formulation the Hill function requires introduction of a currently unknown repressor. Therefore, we propose another function, called the attenuation function *y*_*n*_(*t*) (with the same shape as the Hill function–see S5A Fig and S1 Text), which depends only on time:

$$y_{n}(t)=1-(1-R)\left[1-\frac{1}{T_{1}-T_{2}}(T_{1}exp(-\frac{t}{T_{1}})-T_{2}exp(-\frac{t}{T_{2}}))\right].$$ (1)

Here *R* represents the attenuation coefficient (the smaller *R*, the greater the change of the *k*_*n*_ value and, therefore, the greater impact on the NF-κB system response), while *T*_1_, *T*_2_ are time constants of the putative HS-mediated process. The HS-induced response was incorporated in the model by systematically multiplying different constant rate coefficients *k*_*n*_, by y(t) for the *n*-th process (see S1A Text for the NF-κB model equations). Parameters T_1_ = 6 [min] and T_2_ = 7 [min], affecting the shape of the sigmoidal, have been assumed to be the same for all tested mechanisms and their values have been chosen to obtain a steady state of the attenuation function after 60 min.

To account for heterogeneity in cellular sensitivity to HS, for each simulation, representing a single cell response, *R* was sampled from gamma distribution. The respective parameters (Table 1A) was fitted to obtain a mean value that is smaller than 1, such that the resulting simulations mirror experimental observations with respect to the population-level responses (Fig 1) or fraction of cells exhibiting normal NF-κB responses (Fig 5). This was possible for mechanisms in Fig 3 mechanisms b-e; but not for Fig 3 mechanisms f-i). *R* was replaced by 0 (or by a number 0.001 if the HS-mediated inhibition was modelled by diving appropriate reaction rates by *y*_*n*_) if the sampled value of *R* was smaller than a fitted threshold (0.005 for inhibition of IKK activation and 0.008 for other mechanisms) to account for complete inhibition, observed experimentally in some cells. Following [47, 53] we also assumed that the NF-κB responses depend on the level of IKK protein. To account for varying levels of IKK protein between individual cells, the IKKn production rate was multiplied by the R_IKK_ coefficient, which was sampled from a log-normal distribution with variance σ^2^ = 0.13 and mean μ = 1 (S5B Fig). A number (R_NF-κB_) drawn from the same distribution was used to account for the variability in the total cellular NF-κB level (S4 Fig), by multiplying the nominal level of cytoplasmic NF-κB (10^6^, initialized via NF-κB| IκBα complexes) in each simulated cell.

In order to incorporate the HS effect into the model, the simulation was divided into three phases (generating 1,000 single cell trajectories for each case):

- Phase I: no stimulation to obtain the resting steady state (including randomization for NF-κB and IKK levels).
- Phase II: heat shock and no TNFα. The duration of this phase may vary from 15 to 60 min. During this phase, the specific rate coefficients of the original model change according to the time-dependent attenuation function.
- Phase III: no HS and TNFα stimulation is on. This is the main part of the simulation in which we observe the response of the model with changed parameters.

For the analysis of the IKK depletion, the mathematical model was modified to incorporate a linear term representing the HS-dependent removal of the IKK, corresponding to a transition from a soluble to insoluble fraction (see Fig 6A and S1B Text for model equations). It was assumed that the effect is irreversible (at least in the considered time-scale). Multiple mechanisms were fitted to obtain 80% dampening of the population-level NF-κB responses (see Table 1B for fitted parameters of the gamma distribution for the respective attenuation functions). To fit models involving three HS cross-talk mechanisms (Fig 7, models b*+c+e and b*+d+e), we assumed a 2-fold decrease in the inhibition of the IKK activation or IκB phosphorylation (corresponding to 2-fold increase in the attenuation coefficient *R*, compared to the model b*+c and b*+d). The values of the attenuation coefficient for the NF-κB transport inhibition was then fitted (Table 1). In addition, we assumed that the corresponding attenuation coefficients (i.e. c vs. e, and d vs. e, respectively) are correlated. That is HS simultaneously affected all considered mechanisms per cell. Therefore, Rs have been sampled from the gamma distributions using a random number generator with the same seed but distribution parameters, producing a correlation coefficient equal to 1, S5C Fig).

Simulations were performed in MATLAB R2016a with 'ode23tb' function. Random numbers were generated using Statistics and Machine Learning Toolbox. To distinguish between responding and non-responding cells, hierarchical clustering was performed using 'linkage' function with the built-in method 'ward' (inner squared distance—minimum variance algorithm) and 'euclidean' metric.

### Statistical analyses

Statistical analyses were performed in GraphPad Prism 7.02. Normal distribution was assessed with D’Agostino-Pearson test. Nonparametric tests were applied for non-normal distribution data. Kruskal-Wallis one-way ANOVA with Dunn’s multiple comparisons was used for characteristics of single cell NF-κB responses. Percentage of responding and non-responding cells was assessed with Chi-square test. Spearman’s correlation used to assess relationship between cytoplasmic and nuclear NF-κB p65-EGFP expression in single cells.

## Supporting information

S1 Text NF-κB model equations. (DOCX)

S1 Fig Simulations of individual cross-talk mechanisms. Simulations of mechanisms involved in the NF-κB and HS pathway cross-talk (Fig 3): (a) with no attenuation function; and with attenuation function acting on (b) degradation of IKK (reverse effect), (c) IKK activation, (d) IKK phosphorylation of IκBα, (e) nuclear import of NF-κB, (f) nuclear transport (both ways), (g) transcription, (h) transcription (reverse effect) and (i) translation, for 60 min HS exposure before TNFα stimulation. Shown across considered mechanisms are (**A**) Scatterplots of the maximum nuclear NF-κB level versus the coefficient R_IKK_ corresponding to distributed IKK level per cell. (**B**) Distribution of maximal nuclear NF-κB level in simulated cells.
(TIF)

S2 Fig Analysis of NF-κB p65-Ser536 phosphorylation in transformed cells. The level of p65-Ser536 phosphorylation was analyzed by Western blot in the whole U2OS p65EGFP cell lysates. (**A**) Cells either cultured under normal conditions (37°C) or subjected to 60 min HS at 43°C were treated with TNFα for the indicated times. (**B**) Cells were exposed to 43°C HS for indicated times and subsequently treated with TNFα for 15 min. Shown also are appropriate controls (C denotes no HS no TNFα). β-actin expression was used as a loading control.
(TIF)

S3 Fig Microscopy analyses of single cell NF-κB responses. **(A)** Nuclear NF-κB trajectories in U2OS cells stably expressing p65-EGFP fusion protein (data from Fig 5). Control cells treated with TNFα and cells exposed to 43°C HS for indicated times prior TNFα stimulation. The average depicted with a black line. (B) Correlation between nuclear fluorescence at time t0 and maximum nuclear p65-EGFP (top panel) and between cytoplasmic fluorescence at time t0 and nuclear fluorescence at time t0 (bottom panel) for cells cultured in normal conditions or subjected to 15, 30 and 60 min of HS. Responding cells depicted with yellow circles, non-responding with blue, with fitted regression line and Spearman correlation coefficient (r), respectively. **(C)** Analysis of the normalized single-cell traces of responding cells from Fig 5. Left panel: the distribution of the maximum nuclear p65-EGFP normalized to the fluorescence intensity in the nucleus at time 0. Right panel: the distribution of the maximum nuclear p65-EGFP normalized to the fluorescence intensity in the cytoplasm at time 0. Individual cell data depicted with circles (with mean ± SD per condition). Kruskal-Wallis one-way ANOVA with Dunn’s multiple comparisons test was used (****P value < 0.0001; ns–not significant).
(TIF)

S4 Fig Variable NF-κB levels in the HS cross talk. **(A)** Simulation of HS cross-talk assuming IKK depletion and inhibition of IKK activation (model b*+c from Fig 7) assuming additional distribution of total cellular NF-κB level. Shown are a sample of 50 time courses of simulated nuclear NF-κB levels (colored lines) and average nuclear NF-κB levels (black bold line), calculated from 1,000 single cell simulations for cells treated with TNFα after different HS exposure. **(B)** Percentage (%) of responding (yellow) and non-responding (blue) cells from A. **(C)** Characteristics of NF-κB trajectories in responding cells from B. Left panel: the distribution of the maximum nuclear NF-κB. Right panel: time to first response. **(D)** Scatterplots of the maximum nuclear NF-κB level per cell against (I) attenuation coefficient *R*, (II) IKKn at time t0, (III) IKKn after 60 min HS and (IV) cytoplasmic NF-κB at time t0.
(TIF)

S5 Fig Details of the simulation scheme. **(A)** Comparison between the attenuation function and the Hill function for the repressor (with Hill coefficient η = 1). (**B**) Log-normal distribution used to randomize total cellular IKK and NF-κB levels. (**C**) Correlation between the attenuation coefficients for models incorporating three HS cross-talk mechanisms.
(TIF)
