# Supplementary material for: Quantitative analysis reveals crosstalk mechanisms of heat shock-induced attenuation of NF-κB signaling at the single cell level
Source: PLoS Comput Biol. 2018 Apr 30;14(4):e1006130. doi: 10.1371/journal.pcbi.1006130 (PMC5945226; doi:10.1371/journal.pcbi.1006130)
Supplement: S1 Text — (DOCX) [file pcbi.1006130.s001.docx]

**S1 Text. Model equations and description.**

1. **NF-κB model equations**

| $\frac{d}{dt}IKKn\left( t \right)={R_{IKK}k}_{prod}-{\frac{1}{y_{b}\left( t \right)}k}_{deg}\times IKKn\left( t \right)-T_{R}\times y_{c}\left( t \right)\times k_{1}\times IKKn(t)$ | (1) |
| --- | --- |
| $\frac{d}{dt}IKKa\left( t \right)=T_{R}\times y_{c}\left( t \right)\times k_{1}\times IKKn\left( t \right)-k_{3}\times IKKn\left( t \right)-T_{R}\times k_{2}\times IKKa\left( t \right)\times A20\left( t \right)-\frac{1}{y_{b}\left( t \right)}k_{deg}\times IKKa\left( t \right)-a_{2}\times IKKa\left( t \right)\times I\kappa B\alpha\left( t \right)+t_{1}\times\left( IKKa\vert I\kappa B\alpha\right)\left( t \right)-a_{3}\times y_{d}\left( t \right)\times IKKa\left( t \right)\times\left( I\kappa B\alpha\vert NF\kappa B \right)\left( t \right)+t_{2}\times\left( \mathrm{IKKa}\vert I\kappa B\alpha\vert NF\kappa B \right)\left( t \right)$ | (2) |
| $\frac{d}{dt}IKKi\left( t \right)=k_{3}\times IKKa\left( t \right)+T_{R}\times k_{2}\times IKKa\left( t \right)\times A20\left( t \right)-{\frac{1}{y_{b}\left( t \right)}k}_{deg}IKKi(t)$ | (3) |
| $\frac{d}{dt}\left( IKKa\vert I\kappa B\alpha\right)\left( t \right)=a_{2}\times IKKa\left( t \right)\times I\kappa B\alpha\left( t \right)-t_{1}\times\left( IKKa\vert I\kappa B\alpha\right)\left( t \right)$ | (4) |
| $\frac{d}{dt}\left( \mathrm{IKKa}\left\vert I\kappa B\alpha\right\vert NF\kappa B \right)\left( t \right)=a_{3}\times y_{d}\left( t \right)\times IKKa\left( t \right)\times\left( I\kappa B\alpha\vert NF\kappa B \right)\left( t \right)-t_{2}\times\left( \mathrm{IKKa}\vert I\kappa B\alpha\vert NF\kappa B \right)\left( t \right)$ | (5) |
| $\frac{d}{dt}NF\kappa B\left( t \right)=c_{6a}\times\left( I\kappa B\alpha\vert NF\kappa B \right)\left( t \right)-a_{1}\times I\kappa B\alpha\left( t \right)\times NF\kappa B\left( t \right)+t_{2}\times\left( \mathrm{IKKa}\left\vert I\kappa B\alpha\right\vert NF\kappa B \right)\left( t \right)-i_{1}\times y_{e,f}\left( t \right)\times NF\kappa B\left( t \right)$ | (6) |
| $\frac{d}{dt}{NF\kappa B}_{n}\left( t \right)=i_{1}\times y_{e,f}\left( t \right)\times NF\kappa B\left( t \right)-a_{1}\times k_{v}\times{I\kappa B\alpha}_{n}\left( t \right)\times{NF\kappa B}_{n}\left( t \right)$ | (7) |
| $\frac{d}{dt}A20\left( t \right)=c_{4}\times y_{i}\left( t \right)\times{A20}_{t}(t)-c_{5}\times A20(t)$ | (8) |
| $\frac{d}{dt}{A20}_{t}\left( t \right)={(c}_{2}+c_{1}\times\left( G^{1}+G^{2} \right))\times y_{g}\left( t \right)\times\frac{1}{y_{h}\left( t \right)}-c_{3}\times{A20}_{t}(t)$ | (9) |
| $\frac{d}{dt}I\kappa B\alpha\left( t \right)=-a_{2}\times IKKa\left( t \right)\times I\kappa B\alpha\left( t \right)-a_{1}\times I\kappa B\alpha\left( t \right)\times NF\kappa B\left( t \right)+c_{4a}\times y_{i}\left( t \right)\times{I\kappa B\alpha}_{t}\left( t \right)-c_{5a}\times I\kappa B\alpha\left( t \right)-i_{1a}\times y_{f}\left( t \right)\times I\kappa B\alpha\left( t \right)+e_{1a}\times y_{f}\left( t \right)\times{I\kappa B\alpha}_{n}\left( t \right)$ | (10) |
| $\frac{d}{dt}{I\kappa B\alpha}_{n}\left( t \right)=-a_{1}\times k_{v}\times{I\kappa B\alpha}_{n}\left( t \right)\times{NF\kappa B}_{n}\left( t \right)+i_{1a}\times y_{f}\left( t \right)\times k_{v}\times{I\kappa B\alpha}_{n}\left( t \right)-e_{1a}\times y_{f}\left( t \right)\times{I\kappa B\alpha}_{n}\left( t \right)$ | (11) |
| $\frac{d}{dt}{I\kappa B\alpha}_{t}\left( t \right)={(c}_{2a}+c_{1a}\times\left( G_{a}^{1}+G_{a}^{2} \right))\times y_{g}\left( t \right)\times\frac{1}{y_{h}\left( t \right)}-c_{3a}\times{I\kappa B\alpha}_{t}\left( t \right)$ | (12) |
| $\frac{d}{dt}\left( I\kappa B\alpha\vert NF\kappa B \right)\left( t \right)=a_{1}\times I\kappa B\alpha\left( t \right)\times NF\kappa B\left( t \right)-c_{6a}\times\left( I\kappa B\alpha\vert NF\kappa B \right)\left( t \right)-a_{3}\times y_{d}\left( t \right)\times IKKa\left( t \right)\times\left( I\kappa B\alpha\vert NF\kappa B \right)\left( t \right)+e_{2a}\times y_{f}\left( t \right)\times\left( {{I\kappa B\alpha}_{n}\vert NF\kappa B}_{n} \right)\left( t \right)$ | (13) |
| $\frac{d}{dt}\left( {{I\kappa B\alpha}_{n}\vert NF\kappa B}_{n} \right)\left( t \right)=a_{1}\times k_{v}\times{I\kappa B\alpha}_{n}\left( t \right)\times{NF\kappa B}_{n}\left( t \right)-e_{2a}\times y_{f}\left( t \right)\times\left( {{I\kappa B\alpha}_{n}\vert NF\kappa B}_{n} \right)\left( t \right)$ | (14) |

where:

$G_{a}^{1},G_{a}^{2}$ Logical variables, status of IκBα promoter in first and second homologous gene copy (stochastic)

$G^{1},G^{2}$ Logical variable, status of A20 promoter in first and second homologous gene copy (stochastic)

$T_{R}$ Logical variable, T_R_ = 1 if TNFα signal is present, T_R_ = 0 if no signal is present

$IKKa$ Amount of active form of IKK

$IKKi$ Amount of inactive form of IKK

$IKKn$ Amount of neutral form of IKK

$I\kappa B\alpha$ Amount of free cytoplasmic IκBα

${I\kappa B\alpha}_{n}$ Amount of free nuclear IκBα

${I\kappa B\alpha}_{t}$ Amount of IκBα mRNA transcript

$\mathrm{NF}\kappa B$ Amount of free cytoplasmic NF-κB

${\mathrm{NF}\kappa B}_{n}$ Amount of free nuclear NF-κB

$\left( I\kappa B\alpha|NF\kappa B \right)$ Amount of cytoplasmic IκBα and NF-κB complexes

$\left( {{I\kappa B\alpha}_{n}|NF\kappa B}_{n} \right)$ Amount of nuclear IκBα and NF-κB complexes

$y_{b-i}$ Attenuation functions, where indices b-i refer to the mechanisms described in the main text (only one mechanism tested at a time)

Notation for the remaining complexes is analogous.

Kinetic parameters are taken from [54].

1. **NF-κB model equations for the IKK depletion**

In order to model the IKK transition to the insoluble form, equations (1)-(3) have been modified by adding a linear term representing the loss of IKK. At the same time, the term including $y_{b}$was removed from the model.

| $\frac{d}{dt}IKKn\left( t \right)={R_{IKK}k}_{prod}-k_{deg}\times IKKn\left( t \right)-T_{R}\times y_{c}\left( t \right)\times k_{1}\times IKKn\left( t \right)-{HS\times k}_{insol}\times IKKn\left( t \right)$ | (1) |
| --- | --- |
| $\frac{d}{dt}IKKa\left( t \right)=T_{R}\times y_{c}\left( t \right)\times k_{1}\times IKKn\left( t \right)-k_{3}\times IKKn\left( t \right)-T_{R}\times k_{2}\times IKKa\left( t \right)\times A20\left( t \right)-k_{deg}\times IKKa\left( t \right)-a_{2}\times IKKa\left( t \right)\times I\kappa B\alpha\left( t \right)+t_{1}\times\left( IKKa\vert I\kappa B\alpha\right)\left( t \right)-a_{3}\times y_{d}\left( t \right)\times IKKa\left( t \right)\times\left( I\kappa B\alpha\vert NF\kappa B \right)\left( t \right)+t_{2}\times\left( \mathrm{IKKa}\left\vert I\kappa B\alpha\right\vert NF\kappa B \right)\left( t \right)-{HS\times k}_{insol}\times IKKa\left( t \right)$ | (2) |
| $\frac{d}{dt}IKKi\left( t \right)=k_{3}\times IKKa\left( t \right)+T_{R}\times k_{2}\times IKKa\left( t \right)\times A20\left( t \right)-k_{deg}IKKi\left( t \right)-{HS\times k}_{insol}\times IKKi\left( t \right)$ | (3) |

where:

HS Logical variable, HS = 1 if heat shock signal is present, HS = 0 if no signal is present

$k_{insol}=3.47\cdot{10}^{-4}$ soluble-insoluble transition rate

The rest of parameters and notations remained unchanged.

1. **Comparison between the attenuation function and the Hill function**

A comparison shown on S5 Fig A was obtained with use of a Hill function, given by:

| $h(r(t))=\frac{K^{\eta}}{K^{\eta}+{r(t)}^{\eta}}$ | (1) |
| --- | --- |

where *r(t)* represents repressor protein. The parameters $K=1.56 \cdot{10}^{6}$ and η=1 are interpreted as the value of *r(t)* at which the Hill function reaches half of its maximum value and the cooperativity, respectively. In simulations it was assumed that *r* is a hypothetical protein acting as a repressor whose transcription and translation is described by equations:

| $\frac{d}{dt}r\left( t \right)=k_{r}\times r_{t}\left( t \right)-k_{dr}\times r\left( t \right)$ | (2) |
| --- | --- |
| $\frac{d}{dt}r_{t}\left( t \right)=k_{t}-k_{dt}\times r_{t}\left( t \right)$ | (3) |

where:

$r$ Amount of repressor protein

$r_{t}$ Amount of repressor mRNA transcript

$k_{r}=1.89\cdot{10}^{-3}$ Repressor translation rate

$k_{t}=6.10\cdot{10}^{-3}$ Repressor transcription rate

$k_{dr}=8.96\cdot{10}^{-4}$ Repressor protein degradation rate

$k_{dt}=9.26\cdot{10}^{-4}$ Repressor mRNA degradation rate
