# Supplementary figures and images for: Quantitative analysis reveals crosstalk mechanisms of heat shock-induced attenuation of NF-κB signaling at the single cell level
Source: PLoS Comput Biol. 2018 Apr 30;14(4):e1006130. doi: 10.1371/journal.pcbi.1006130 (PMC5945226; doi:10.1371/journal.pcbi.1006130)

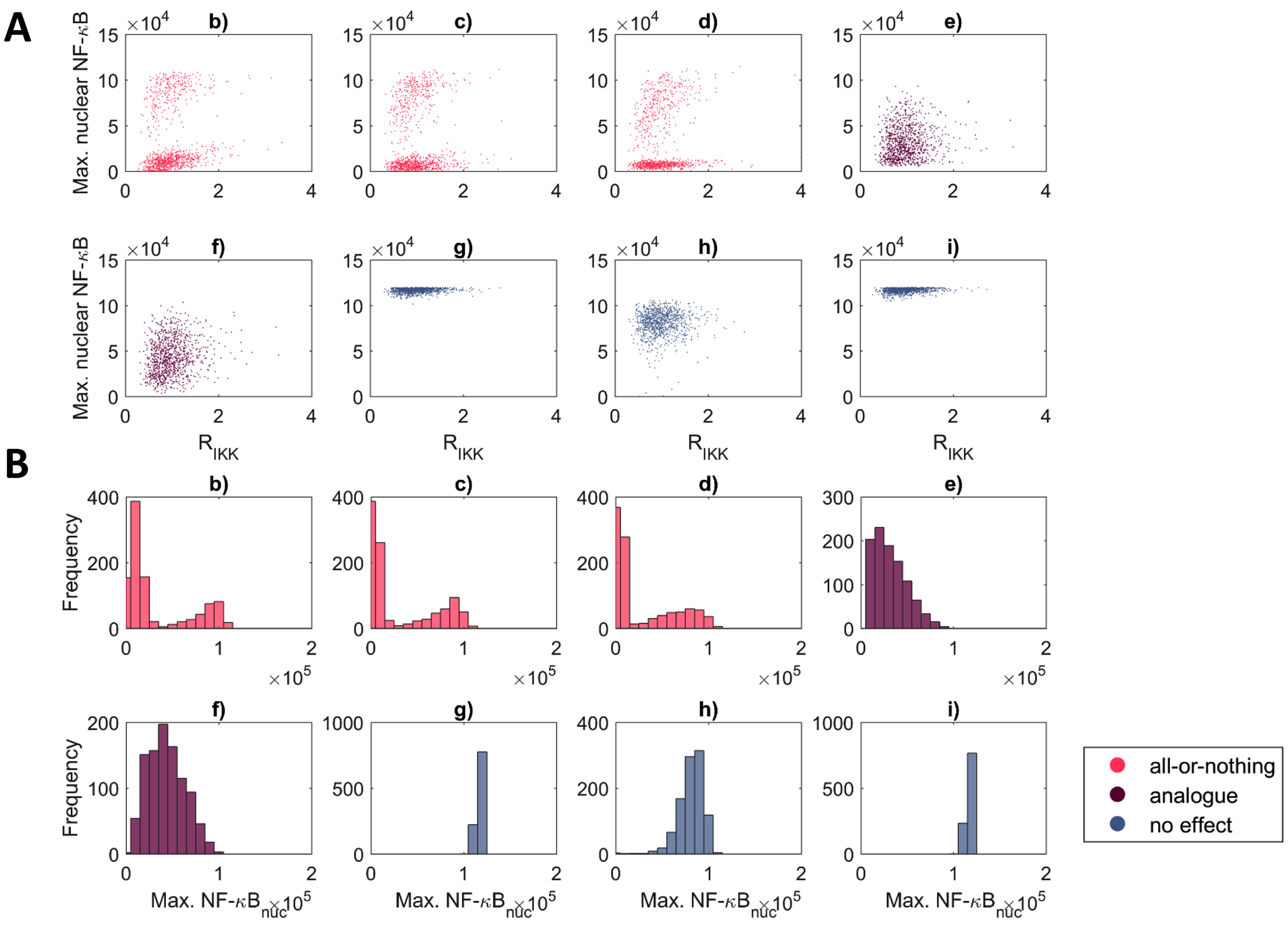

Supplement: S1 Fig — Simulations of mechanisms involved in the NF-κB and HS pathway cross-talk (Fig 3): (a) with no attenuation function; and with attenuation function acting on (b) degradation of IKK (reverse effect), (c) IKK activation, (d) IKK phosphorylation of IκBα, (e) nuclear import of NF-κB, (f) nuclear transport (both ways), (g) transcription, (h) transcription (reverse effect) and (i) translation, for 60 min HS exposure before TNFα stimulation. Shown across considered mechanisms are (A) Scatterplots of the maximum nuclear NF-κB level versus the coefficient RIKK corresponding to distributed IKK level per cell. (B) Distribution of maximal nuclear NF-κB level in simulated cells. (TIF) [file pcbi.1006130.s002.tif]

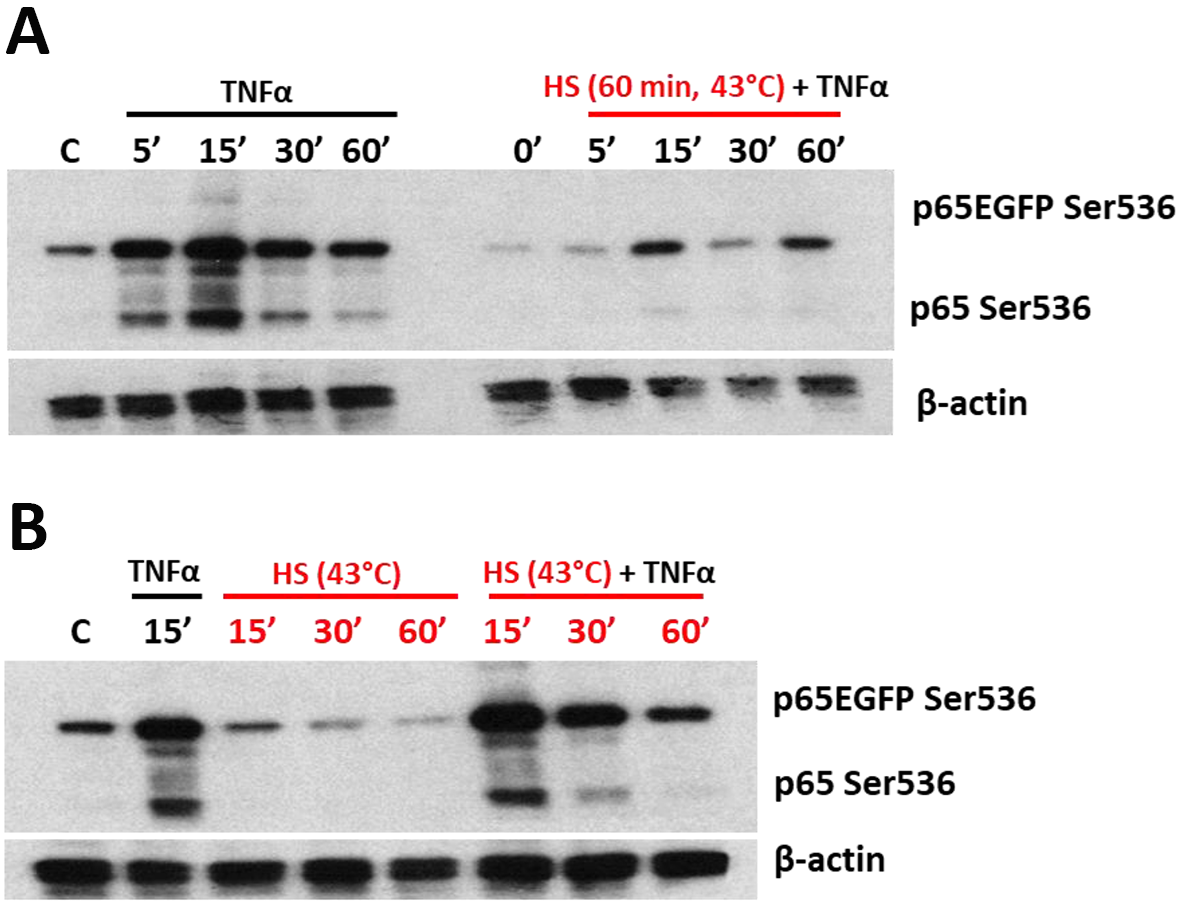

Supplement: S2 Fig — The level of p65-Ser536 phosphorylation was analyzed by Western blot in the whole U2OS p65EGFP cell lysates. (A) Cells either cultured under normal conditions (37°C) or subjected to 60 min HS at 43°C were treated with TNFα for the indicated times. (B) Cells were exposed to 43°C HS for indicated times and subsequently treated with TNFα for 15 min. Shown also are appropriate controls (C denotes no HS no TNFα). β-actin expression was used as a loading control. (TIF) [file pcbi.1006130.s003.tif]

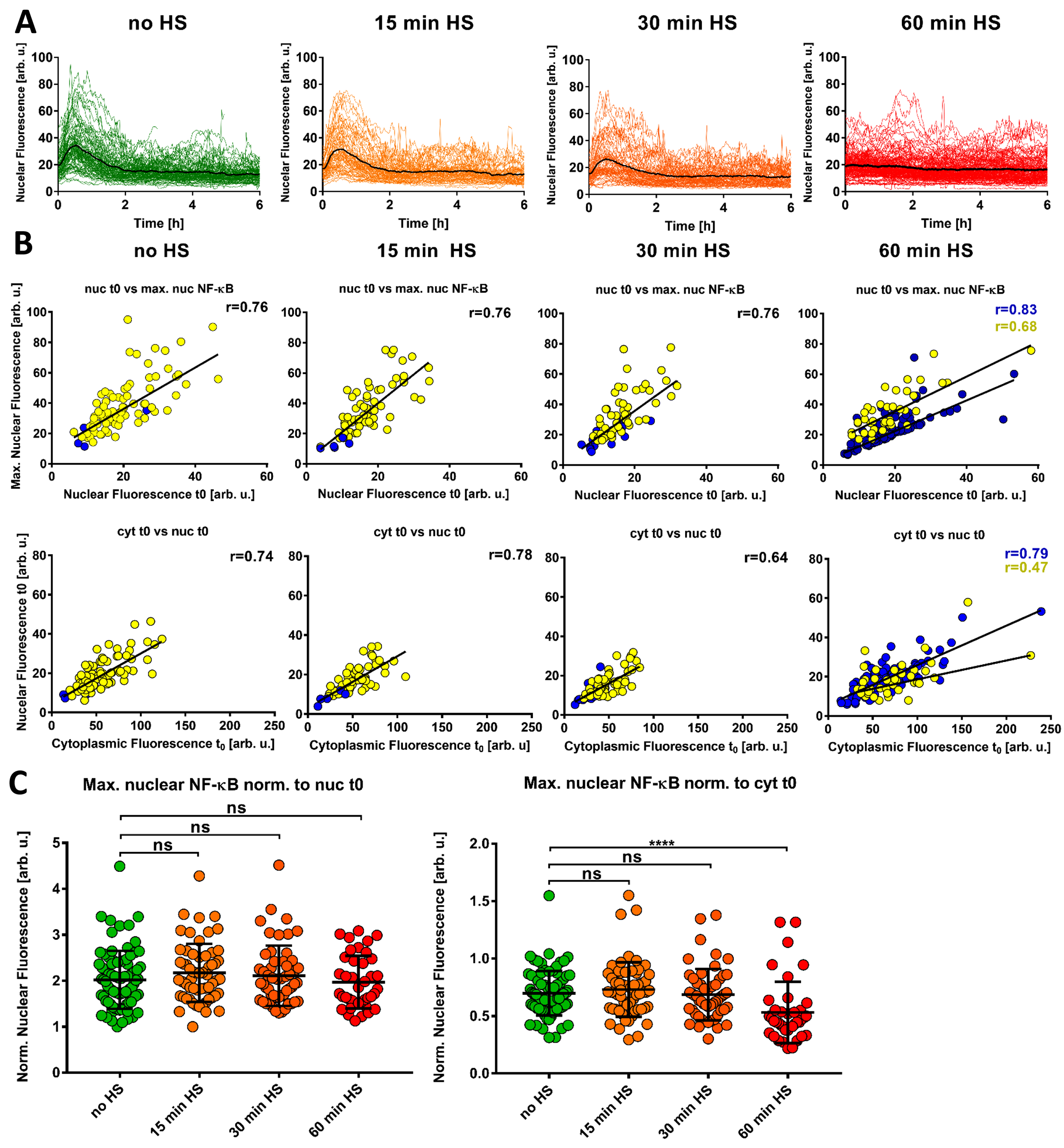

Supplement: S3 Fig — (A) Nuclear NF-κB trajectories in U2OS cells stably expressing p65-EGFP fusion protein (data from Fig 5). Control cells treated with TNFα and cells exposed to 43°C HS for indicated times prior TNFα stimulation. The average depicted with a black line. (B) Correlation between nuclear fluorescence at time t0 and maximum nuclear p65-EGFP (top panel) and between cytoplasmic fluorescence at time t0 and nuclear fluorescence at time t0 (bottom panel) for cells cultured in normal conditions or subjected to 15, 30 and 60 min of HS. Responding cells depicted with yellow circles, non-responding with blue, with fitted regression line and Spearman correlation coefficient (r), respectively. (C) Analysis of the normalized single-cell traces of responding cells from Fig 5. Left panel: the distribution of the maximum nuclear p65-EGFP normalized to the fluorescence intensity in the nucleus at time 0. Right panel: the distribution of the maximum nuclear p65-EGFP normalized to the fluorescence intensity in the cytoplasm at time 0. Individual cell data depicted with circles (with mean ± SD per condition). Kruskal-Wallis one-way ANOVA with Dunn’s multiple comparisons test was used (****P value < 0.0001; ns–not significant). (TIF) [file pcbi.1006130.s004.tif]

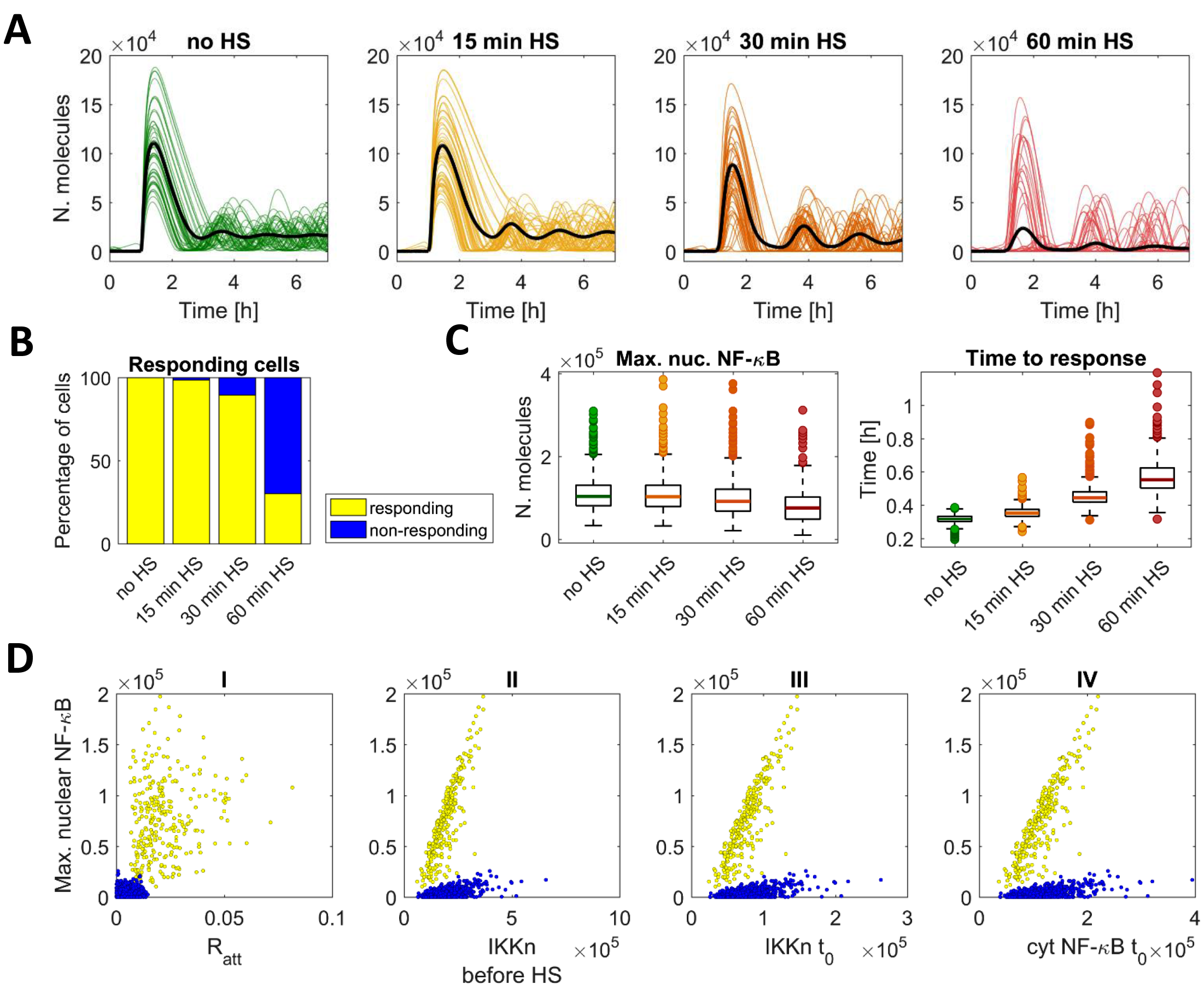

Supplement: S4 Fig — (A) Simulation of HS cross-talk assuming IKK depletion and inhibition of IKK activation (model b*+c from Fig 7) assuming additional distribution of total cellular NF-κB level. Shown are a sample of 50 time courses of simulated nuclear NF-κB levels (colored lines) and average nuclear NF-κB levels (black bold line), calculated from 1,000 single cell simulations for cells treated with TNFα after different HS exposure. (B) Percentage (%) of responding (yellow) and non-responding (blue) cells from A. (C) Characteristics of NF-κB trajectories in responding cells from B. Left panel: the distribution of the maximum nuclear NF-κB. Right panel: time to first response. (D) Scatterplots of the maximum nuclear NF-κB level per cell against (I) attenuation coefficient R, (II) IKKn at time t0, (III) IKKn after 60 min HS and (IV) cytoplasmic NF-κB at time t0. (TIF) [file pcbi.1006130.s005.tif]

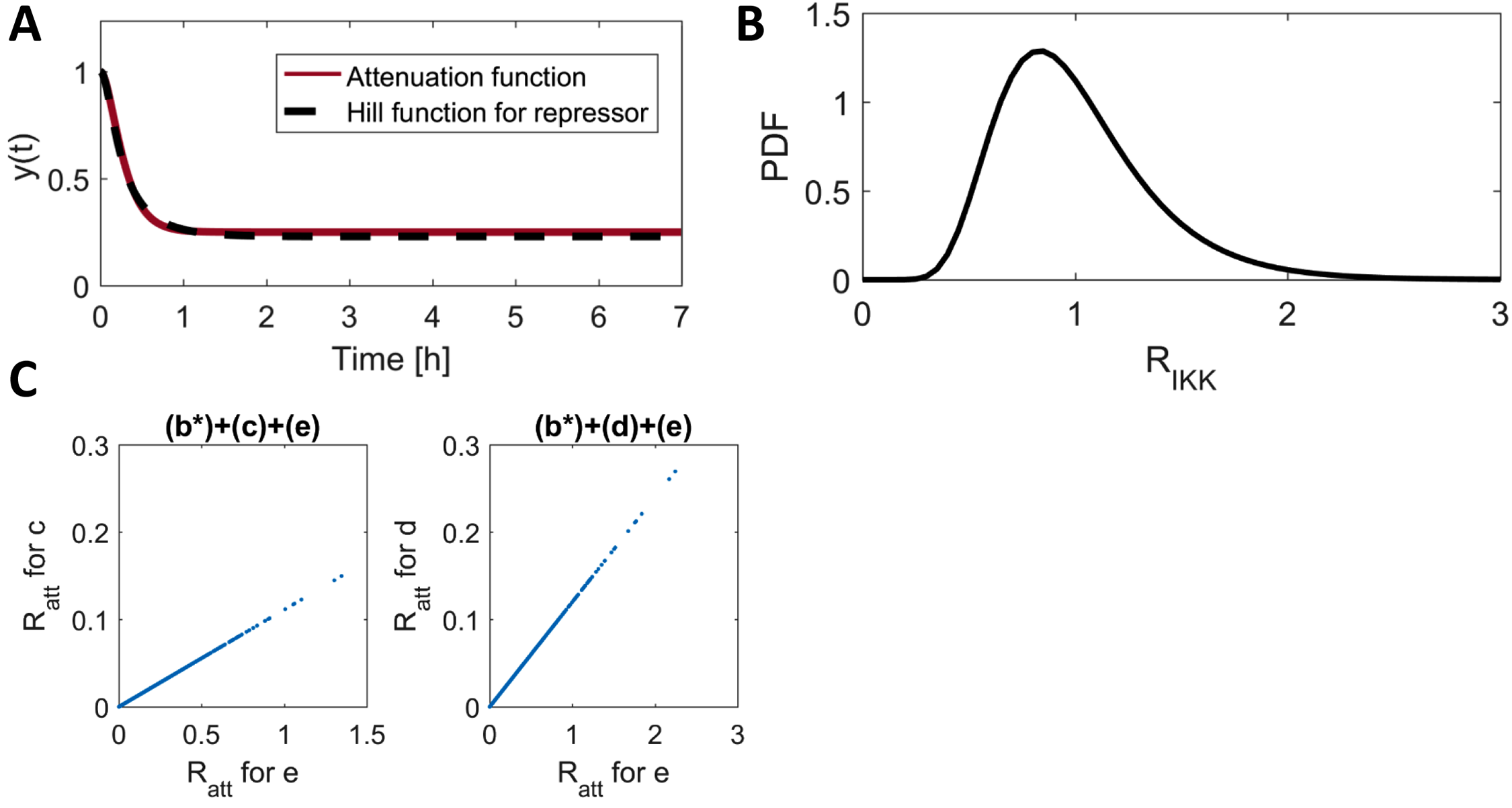

Supplement: S5 Fig — (A) Comparison between the attenuation function and the Hill function for the repressor (with Hill coefficient η = 1). (B) Log-normal distribution used to randomize total cellular IKK and NF-κB levels. (C) Correlation between the attenuation coefficients for models incorporating three HS cross-talk mechanisms. (TIF) [file pcbi.1006130.s006.tif]
